# Adaptations of Interferon Regulatory Factor 3 with Transition from Terrestrial to Aquatic Life

**DOI:** 10.1038/s41598-020-61365-9

**Published:** 2020-03-11

**Authors:** Monica Angeletti, Wan-Ling Nicole Hsu, Nashaat Majo, Hideaki Moriyama, Etsuko N. Moriyama, Luwen Zhang

**Affiliations:** 1 School of Biological Sciences, University of Nebraska, Lincoln, NE 68588 USA; 2 Center for Plant Science Innovation, University of Nebraska, Lincoln, NE 68588 USA; 3 Nebraska Center for Virology, University of Nebraska, Lincoln, NE 68583 USA; 4 Present Address: Department of Biostatistics, University of Washington, Washington, USA

**Keywords:** Innate immunity, Immunology, Evolution, Molecular evolution

## Abstract

Interferon regulatory factor 3 (IRF3) and IRF7 are closely related IRF members and the major factors for the induction of interferons, a key component in vertebrate innate immunity. However, there is limited knowledge regarding the evolution and adaptation of those IRFs to the environments. Two unique motifs in IRF3 and 7 were identified. One motif, GASSL, is highly conserved throughout the evolution of IRF3 and 7 and located in the signal response domain. Another motif, DPHK, is in the DNA-binding domain. The ancestral protein of IRF3 and 7 seemed to possess the DPHK motif. In the ray-finned fish lineage, while the DPHK is maintained in IRF7, the motif in IRF3 is changed to NPHK with a D → N amino acid substitution. The D → N substitution are also found in amphibian IRF3 but not in amphibian IRF7. Terrestrial animals such as reptiles and mammals predominantly use DPHK sequences in both IRF3 and 7. However, the D → N substitution in IRF3 DPHK is again found in cetaceans such as whales and dolphins as well as in marsupials. These observations suggest that the D → N substitutions in the IRF3 DPHK motif is likely to be associated with vertebrate’s adaptations to aquatic environments and other environmental changes.

## Introduction

The innate immune system comprises the cells and the mechanisms that defend the host from infection by other organisms in a non-specific manner. It is present in all classes of life. Type I interferons (IFNs) are proteins made and released by host cells in response to the presence of pathogens—such as viruses, bacteria, parasites, or tumor cells. They are a key component in the vertebrate innate response to invading pathogens. Cells use many sensor molecules to recognize pathogen-associated molecular patterns and initiate innate as well as adaptive immune responses against pathogens^1–4^.

**Figure 1:**
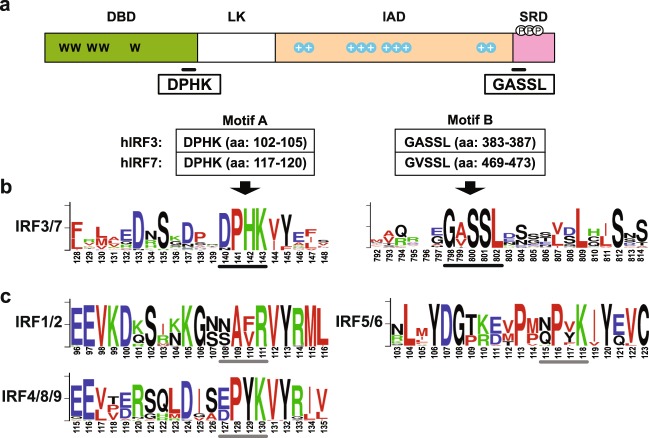
Identification of two conserved motifs unique to the IRF3/7 proteins. (**a**) Schematic diagram of an IRF3/7 protein. The DNA-binding domain (DBD), the IRF-association domain (IAD), the signal response domain (SRD), as well as the linker region (LK) are shown in different colors. Five well conserved tryptophans (W) in DBD, clusters of positively charged amino acids (+) located in IAD, and phosphorylation sites (P) in SRD are also shown. The amino acid positions of the two motifs (A and B) in the human IRF3 (hIRF3) and 7 (hIRF7) proteins are based on Q14653 and Q92985, respectively. (**b**) Conserved amino acids found in the two motif regions of IRF3/7 proteins. Sequence logos are used to illustrate the amount of sequence conservation for each position. The overall height of the stack of letters indicates the sequence conservation at each position. The height of symbols within each stack indicates the relative frequency of each amino acid. The multiple sequence alignment was generated using 12 IRF3 and 13 IRF7 protein sequences listed in Supplementary Table S1. The position numbers are based on the multiple sequence alignment (see Supplementary Fig. S2 for the sequence logo for the entire alignment). The two motifs (A and B) are indicated with thick black bars under the logo. Following colors are used for different amino acids: green (K, R, H), blue (D and E), red (A, V, L, I, P, W F, M), and black for all others. (**c**) Conserved amino acid patterns in other IRF families. Sequence logos were generated from the region corresponding to the motif A. For each IRF subfamily, multiple alignment was generated using 13 IRF protein sequences listed in Supplementary Table S1. The amino acid positions corresponding to the motif A found in the IRF3 and 7 are marked with gray bars under the logo. All sequence logos were generated using WebLogo v2.8.2 (https://weblogo.berkeley.edu)^63^.

IFN regulatory factors (IRFs) are a small family of transcription factors. The member proteins share extensive similarity in the DNA-binding domain (DBD) located in the N-terminus, which is characterized by a series of five well-conserved tryptophans (Ws in Fig. 1a). The DBD region contains a helix-turn-helix structure and recognizes a DNA sequence known as IFN-stimulated response elements^5^. The C-terminal portion of IRFs is variable and defines their specific biological functions. The IRF family has a variety of functions including, but not limited to, apoptosis, oncogenesis, host defense, and viral latency^6–10^.

IRF3 and 7 are the two critical IRF members for IFN production^10–14^. They share structural and sequence similarities and are considered to be originated from a common ancestral IRF gene. In addition to the N-terminal DBD, the C-terminal region of these proteins contain the IRF-association (IAD) and signal response domains (or serine-rich, SRD) (Fig. 1a). These proteins need to be activated for their functions. Normally IRF3 and 7 stay in the cytoplasm. Transcriptional activity of IRF3 or 7 is controlled by virus and other pathogenic stresses that initiate phosphorylation events in the C-terminal SRD. Those phosphorylation events are mediated by several kinases. It enables IRF3 and 7 to form homo- or hetero-dimers, move from cytoplasm to nucleus, bind to target DNAs, and activate transcription of target genes^6–10,15^. Albeit exciting findings have been made since their discovery, evolutionary studies of IRF3 and 7 have been limited^16–19^.

Vertebrate species have experienced significant environmental transitions during their evolution. Among the most drastic changes happened when the ancestral aquatic vertebrates emerged to land and adapted to terrestrial environments. The secondary transition that happened in some species from terrestrial to aquatic environments was equally significant. These environmental transitions presented numerous challenges to ancestral vertebrates, and necessitated adaptations in various anatomies and biological processes including innate immune systems^20,21^. To study how these genes responded to environmental transitions during vertebrate evolution, we collected IRF3 and 7 protein sequences from all available vertebrate species and examined how their sequences changed during evolution. We identified two motifs uniquely conserved in the IRF3/7 subfamily: one motif, GASSL, at the start of the SRD and another motif, DPHK, at the C-terminal edge of the DBD. While the DPHK motif in IRF7 is largely conserved throughout the vertebrate evolution, D → N substitutions at the first position of the IRF3 motif appear to have happened in a limited number of different lineages independently. The D → N substitutions are apparently associated with the evolution of ray-finned fish and amphibian lineages. In land animals, the D → N substitutions in IRF3 were found only in marsupials and cetaceans (whales and dolphins). Because the D → N substitutions in IRF3 DPHK are mainly associated with animals adapted to aquatic environments (ray-finned fish, amphibians, and cetaceans), the NPHK motif in IRF3 proteins may play a role in their adaptive evolution during land-water transitions.

## Results

### Identification of conserved motifs unique to IRF3 and IRF7 proteins

The IRF family has nine members in humans^8,9^. Consistent to the previous findings, our preliminary phylogenetic analysis among 116 representative IRF proteins grouped them into four well-supported major subfamilies: IRF1/2, IRF3/7, IRF5/6, and IRF4/8/9 groups (Supplementary Fig. S1 and Supplementary Table S1). Among the four subfamilies, the IRF3/7 subfamily is the most divergent. As shown in Supplementary Fig. S2, the N-terminal DBD area, including the five Ws, is clearly highly conserved. Excluding this highly conserved area, only two motifs that include at least three consecutively conserved amino acids were identified: DPHK and GASSL (marked with black dots in Supplementary Fig. S2).

Although the IRF DBD region is highly conserved, the DPHK motif is found only in IRF3/7 (Fig. 1b,c). Comparison of the 116 representative protein sequences including all IRF subfamilies confirmed that while many amino acid positions are completely conserved among all IRF groups, the DPHK motif is not (Supplementary Fig. S3). We further verified the conservation of the DPHK motif in much larger datasets including more than 100 each of IRF3 and IRF7 proteins (Supplementary Tables S2 and S3). When the IRF3 and IRF7 protein sequences were compared separately, we noted that the first amino acid of the motif, aspartic acid (D), is not as strongly conserved in IRF3 (Supplementary Fig. S4a) as in IRF7 (Supplementary Fig. S4b).

Another motif (GASSL) is identified to be uniquely conserved in IRF3 and 7, but not in any other IRF proteins (Fig. 1b). As shown in Supplementary Fig. S5, variations are found in the second position of the motif depending on IRF3 or IRF7. While the motif GASSL is completely conserved in the IRF3 proteins (Supplementary Fig. S5a), in IRF7, alanine (A) and valine (V) are found almost equally at the second position of the motif (Supplementary Fig. S5b).

### Ancestral sequences of the IRF3 and 7 motifs

**Figure 2:**
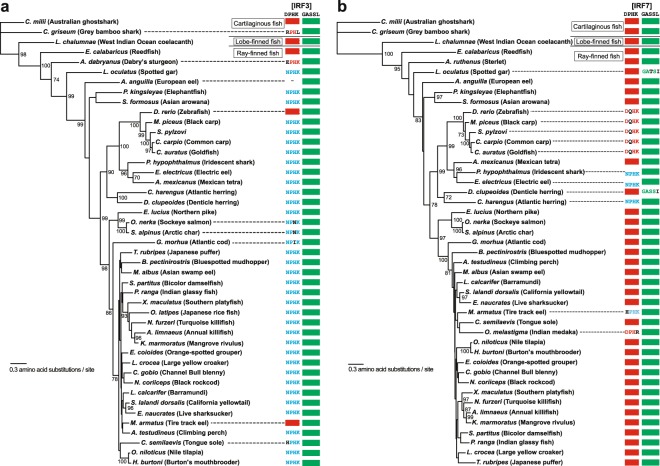
Distribution of IRF3 and 7 motifs in fish. The maximum-likelihood phylogenies of IRF3 (**a**) and IRF7 (**b**) proteins are shown with the bootstrap supporting values (%) at the nodes where the supporting values are equal to or higher than 70. Shark sequences are used as the outgroups. When the motif sequence for a species is DPHK or GASSL, it is shown with a red or green closed box, respectively. When the motif sequence is NPHK, it is shown with blue fonts. Any other amino acid changes in the motif sequences are shown in black fonts. See Supplementary Table S4 for the protein sequences used. The visualization of the phylogenies was performed using FigTree v1.4.4 (http://tree.bio.ed.ac.uk/software/figtree/).

IRF3 and 7 have not been identified in Cephalochordata (such as lancelets) nor in Urochordata and are considered as vertebrate-specific IRFs^16,18,19,22,23^. Furthermore, the sole IRF protein characterized so far from a lamprey (Agnatha, jawless fishes) is closely related to the IRF1/2-group proteins^19,24^. Within the Gnathostomata (jawed vertebrates), cartilaginous fishes (sharks and rays) diverged first at ~450 million-years ago (MYA)^25^. IRF3 and 7 were both identified in the two shark species where sequences are publicly available. While the two motifs (DPHK and GASSL) were found in the IRF3 and IRF7 of Australian ghost shark (or elephant shark, *Callorhinchus milii*), the DPHK motif of IRF3 is changed to RPHL in grey bamboo shark (*Chiloscyllium griseum*) (Fig. 2 and Supplementary Table S4).

Considering that IRF3/7-group proteins are found only in jawed vertebrates including sharks, the ancient duplication event that generated IRF3 and 7 proteins must have happened before the divergence of the shark lineages. Considering also that, except for the *C. griseum* IRF3, the DPHK and GASSL motifs are conserved from sharks and coelacanths (lobe-finned fish) to reedfish (Polypteriformes, the most ancient group of ray-finned fish that have functional lungs and morphological traits comparable to the Late Devonian lobe-finned fishes)^26,27^ (Fig. 2), DPHK and GASSL appear to be the ancestral sequences of these motifs.

### Preference of NPHK over DPHK in ray-finned fish IRF3

**Figure 3:**
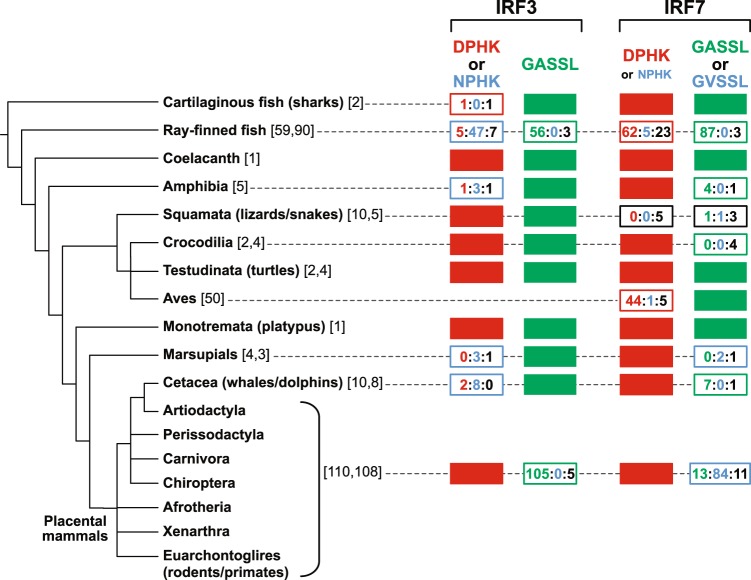
Summary of the distribution of IRF3 and 7 motifs in vertebrates. The evolutionary relationships among vertebrates are a composite of multiple studies^66^. The numbers in square brackets after each vertebrate group name show the number of IRF3 proteins examined followed by the one for IRF7 (when the number is different). When the motif sequence for a given vertebrate group is completely conserved with DPHK or GASSL, it is shown with a red or green closed box, respectively. When the motif sequence varies, the numbers of occurrences are shown in each box as follows: for the motif A, DPHK in red, NPHK in blue, and any other sequences in black; for the motif B, GASSL in green, GVSSL in blue, and any other sequences in black. See Supplementary Tables S2–S5 for the protein sequences used.

Ray-finned fish have a complete and highly specialized type I IFN system^28,29^. To examine the evolution of the two motifs during fish evolution, IRF3 and 7 sequences from various fish species (62 and 93 sequences, respectively) were collected (Supplementary Table S4). Bony fishes (Osteichthyes) are divided into ray-finned fish (Actinopterygii) and lobe-finned fish (Sarcopterygii). As mentioned above, the DPHK and GASSL motifs are conserved in sharks as well as in coelacanth (lobe-finned fish) for both IRF3 and IRF7. Within the ray-finned fish lineage, both of these motifs are still perfectly conserved in reedfish (Fig. 2). Furthermore, throughout the ray-finned fish evolution, the GASSL motif is almost completely conserved both in IRF3 and 7 (Figs. 2b and 3). On the contrary, the DPHK motif has been changed both, but at different degrees, in IRF3 and IRF7. In IRF3, almost the entire ray-finned fishes have NPHK instead of DPHK for the motif indicating that the substitution from aspartic acid (D) to asparagine (N) must have happened at the first amino acid of the DPHK motif before the divergence of the main ray-finned fish linage (Figs. 2a and 3, and Supplementary Tables S4). Back mutations to DPHK were found only in a few cases (such as in *Danio rerio*). In contrast, the DPHK motif is maintained in the majority of the fish IRF7, with D → N substitutions found only in a limited number of lineages. Therefore, selective constraints to maintain the DPHK motif in the two IRF proteins do not seem to be the same during the ray-finned fish evolution.

### NPHK motif are used in amphibian IRF3

**Figure 4:**
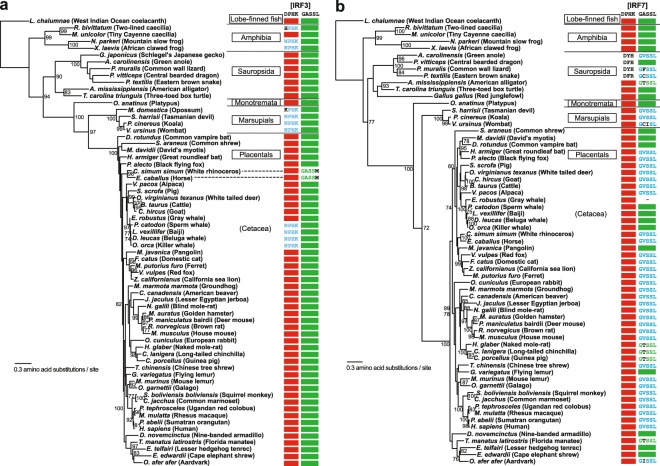
Distribution of IRF3 and 7 motifs in amphibians, reptiles, and mammals. The maximum-likelihood phylogenies of IRF3 (**a**) and IRF7 (**b**) proteins are shown with the bootstrap supporting values (%) at the nodes where the supporting values are equal to or higher than 70. The coelacanth (*Latimeria chalumnae*) sequences are used as the outgroups. When the motif sequence for a species is DPHK or GASSL, it is shown with a red or green closed box, respectively. When the motif sequence is NPHK (for motif A) or GVSSL (for motif B), it is shown with blue fonts. Any other amino acid changes in the motif sequences are shown in black fonts. See Supplementary Tables S2 and S3 for the protein sequences used. The visualization of the phylogenies was performed using FigTree v1.4.4 (http://tree.bio.ed.ac.uk/software/figtree/).

We further examined IRF3 and 7 protein sequences from land animals. While in all five amphibian IRF7 proteins we examined, the motif A is conserved with DPHK, the sequence was changed in four amphibian IRF3 proteins with three having NPHK sequences. The GASSL motif is conserved in all of five amphibian IRF3 and 7 proteins (Figs. 3 and 4, and Supplementary Table S3).

In the 14 reptilian species, IRF3 motifs are completely conserved with DPHK and GASSL sequences. The major groups of reptiles are Squamata (lizards and snakes), Testudines (turtles), and Crocodilia (crocodiles and alligators). Interestingly, within the 13 species, IRF7 motifs vary in the squamate lineage including the possible loss of the DPHK motif (Figs. 3 and 4, and Supplementary Table S3). It is thus possible that the squamates may have functional IRF3 proteins but inefficient or non-functional IRF7 proteins in their innate immune systems. Other reptiles (including both crocodilians and testudines) seem to have maintained fully functional ancestral-type IRF3 and 7 proteins. While birds are closely related to crocodilians, as reported previously^18,30^, we did not identify IRF3 proteins from birds. However, in IRF7 proteins from 50 species of birds, the two motifs (DPHK and GASLL) are highly conserved (Fig. 3, Supplementary Fig. S6, and Supplementary Table S6).

Considering that sharks and coelacanth have the DPHK motif in IRF3, we can infer the direction of the amino acid substitution found at the first position of this motif to be from D to N in the amphibian lineage. Since reptiles maintain the ancestral DPHK motif in IRF3, the same D → N evolution found in the IRF3 motif seem to have happened independently in two different animal lineages, ray-finned fish and amphibians.

### NPHK motif in IRF3 are only used in two mammalian lineages

Mammals are divided into two major groups: Monotremata and Theria. Therian mammals include marsupials and placental mammals. The platypus (*Ornithorhynchus anatinus*) is one of the five extant species of monotremes, the only mammals that lay eggs instead of giving birth to live young. Platypus has conserved DPHK and GASSL motifs in both IRF3 and 7 (Figs. 3 and 4, and Supplementary Table S2). Similarly, almost all of 125 mammalian species we examined had conserved DPHK and GASSL motifs in IRF3 proteins. Mammalian IRF7 proteins (from 120 species), however, have a preference of GVSSL over the GASSL (Figs. 3 and 4, and Supplementary Table S2). There are two noteworthy exceptions in the DPHK motif in mammalian IRF3.

Cetaceans are a group of aquatic mammals, which include whales, dolphins, and porpoises. Although originated from land mammals (~55 MYA), they successfully readapted to aquatic environments^31,32^. Interestingly, eight out of ten cetacean species we examined use NPHK sequences in their IRF3 (Figs. 3 and 4a, and Supplementary Table S2). DPHK is used in all cetacean IRF7 examined. The GASSL motif is also well conserved in both IRF3 and 7 proteins in cetaceans (Fig. 4).

Another exception was found in marsupials, a unique group of mammals whose neonates are born incompletely developed and are typically carried and suckled in a pouch. They also have the NPHK motif in IRF3 (Fig. 4a and Supplemental Table S2).

As the monotreme (platypus) and the majority of the mammals use the DPHK sequence in their IRF3, the direction of the amino acid substitutions found in marsupials and cetaceans is clearly from D to N. It is interesting to note that the same D → N substitutions in the IRF3 motif were found in animals adapted to aquatic life (ray-finned fish, amphibians, and cetaceans).

### Structural analysis of IRF3 and IRF7 proteins

**Figure 5:**
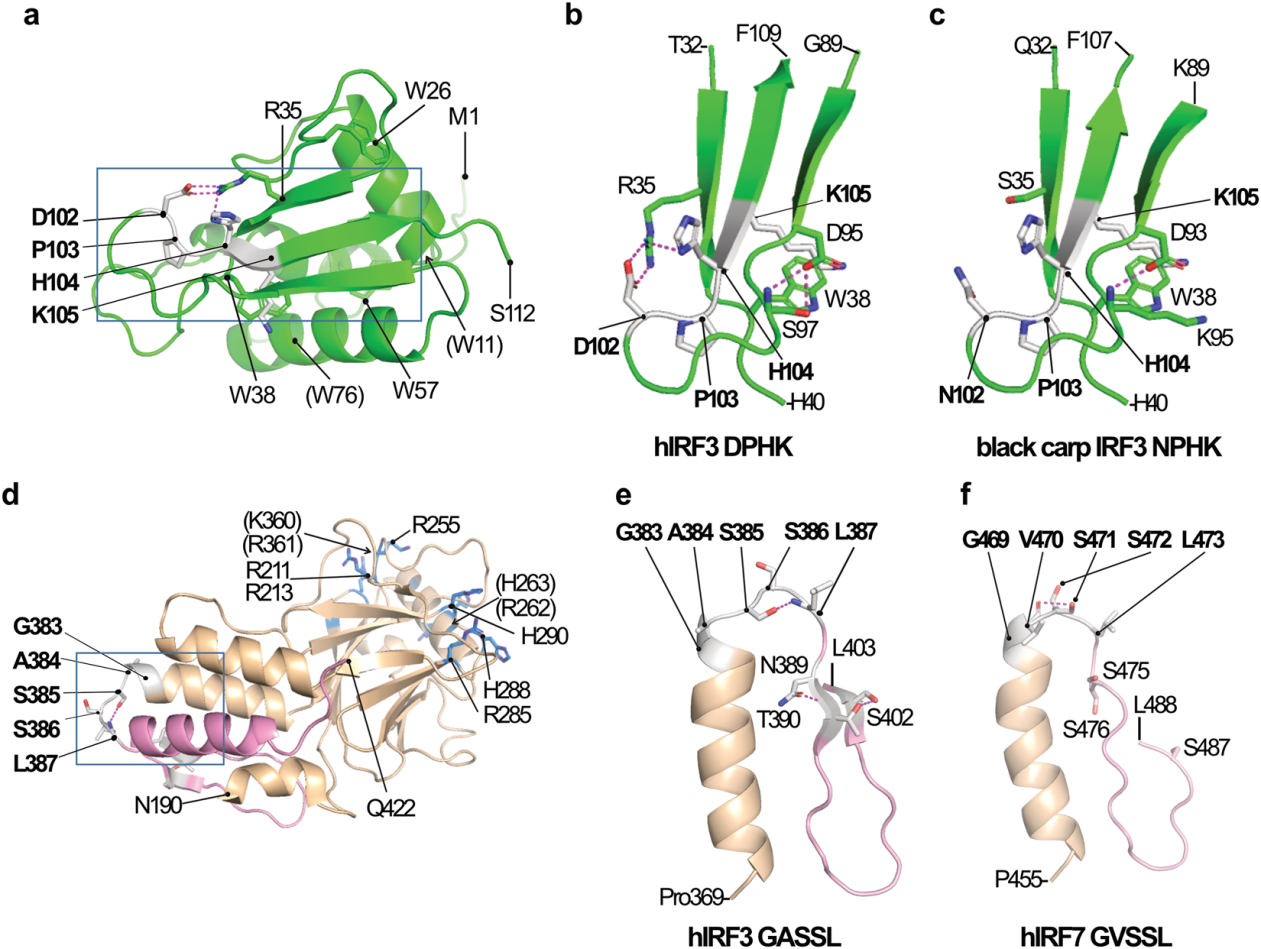
Three-dimensional structures of IRF3 and 7. (**a**) The 3D-structure of the human IRF3 (hIRF3) DBD (PDB 2O6G, from M1 to S112). Residues in the DPHK signature are shown in white with side chains in stick models. Possible hydrogen bonds are represented by dotted magenta lines. Conserved W residues involved in DNA binding are shown in sticks. The boxed area is enlarged in (**b**). Residues indicated in parentheses are located at the back side of the drawing. (**b**) The 3D-structure of the DPHK motif area. The part of the hIRF3 protein structure (boxed in **a**) including the DPHK motif area (G89-F109) and interacting amino acids (T32-H40) are shown. (**c**) The modeled 3D-structure of the NPHK motif area in the black carp IRF3. The structure was modeled based on the hIRF3 structure (PDB 2PI0). (**d**) The 3D-structure of the hIRF3 C-terminal region (PDB 2O6G, from D190 to Q422). The IAD and SRD regions are shown in wheat and pink, respectively. The residues in the GASSL motif is shown in white with side chains in stick models. Ten conserved positively charged residues (R, K, and H) involved in the attachment to adaptors are shown in blue. The boxed area is enlarged in (**e**). Residues indicated in parentheses are located at the back side of the drawing. (**e**) The 3D-structure of the GASSL motif area. The part of the hIRF3 protein structure (boxed in **d**) including the GASSL motif area (P369-L403) are shown. (**f**) The modeled 3D-structure of the GVSSL motif area in the hIRF7. The structure was modeled using the hIRF7 sequence (Q92985, P455-L488) with the hIRF3 structure (PDB 1QWT) as the template. While N389, T390, and S402 in hIRF3 are on β-strands (**e**), corresponding positions in hIRF7 (S475, S476, and S487) were predicted to be in the loop structure. Structural mining and preparation of graphics were performed using the PyMOL Molecular Graphics System, version 2.3.2 (Schrödinger, LLC, New York, NY, USA; https://pymol.org/).

To understand how the amino acid substitutions observed in the motif and surrounding sequences affected the functions of the IRF3 and 7 proteins, we examined the three-dimensional (3D) structures of these proteins. The 3D-structures of the human IRF3 (hIRF3) protein are available for the DBD (with the DPHK motif, Fig. 5a)^33^ and for the C-terminal region (with the GASSL motif, Fig. 5d)^34^. We generated the models of the 3D-structures for the IRF3 DBD from the black carp (*Mylopharyngodon piceus*) with the NPHK motif and for the human IRF7 (hIRF7) C-term region with the GVSSL motif.

In the hIRF3 DBD, D102 (the first amino acid of the DPHK motif) is on the loop structure between two β-strands (β3 and β4, Supplementary Fig. S4) and exposed to solvent (Fig. 5b). P103 and the neighboring main chain provide a hydrophobic pocket to guide DNA binding^35^. H104 is also in the solvent side and takes a trans-position against K105. When IRF3 binds to DNA, K105 binds to the phosphate atom of the DNA with W38 and R81 (see also Supplementary Fig. S4). The orientation of K105 is determined by the local hydrogen-bond network, D102–R35–H104, because H104 and K105 are located in the same β-strand (β4) (Fig. 5a,b).

In the 3D-structure model of the black carp IRF3 DBD, NPHK retained the chain trace as DPHK (Fig. 5c). The D102-R35-D104 hydrogen-bond network found in hIRF3 is lost providing more freedom in the local structure. Nevertheless, DNA-binding activity is likely to be retained since the replacement of S97 (in hIRF3) by K95 maintained the hydrogen bond with D93 (D95 in the hIRF3), which prevents the contact between K105 and D93 (such a contact could interfere DNA-binding). With the substitution from D102 to N102, a negative charge is removed and a positive charge is introduced in the solvent side. This might affect the phosphorylation-induced activation process of IRF3 and the deployment of the DBD from the acidic C-terminal region^34^.

In the hIRF3, the GASSL motif is located in the loop between an α-helix (α4) and a β-strand (β12) (positions 383–387; Fig. 5d,e, and Supplementary Fig. S5). The ten positively charged residues (Rs and Ks) that were identified to form the basic surface and considered to involve in stabilizing the acidic C-terminal tail upon phosphorylation are shown in Fig. 5d (see also Supplementary Fig. S5a)^34^. G383 and A384 are likely involved in helical capping to stabilize the loop-stem structure. S385 is hydrogen bonded to L387 and provides a constrain in the SRD (colored in pink). S386 is exposed to solvent and ready for phosphorylation^36^. Contraction of the SRD is also held by the hydrogen-bond network, N389–T390–S402 (Fig. 5e). Phosphorylation of S386 exchanges the hydrogen bond from S385–L387 to A384–S385, which relaxes the constrain of the SRD allowing dimerization of IRF3 and hence, DNA binding^36,37^.

In the hIRF7 3D-structure model with the GVSSL motif (Fig. 5f), the main-chain trace was the same as in the hIRF3 with GASSL (Fig. 5e). However, by replacement of A with V, the bulkiness of the hydrophobic residue increased in correspondence to two carbon atoms (Fig. 5f). A hydrogen bond formed between V470 and S471 (instead of S385-L387 hydrogen bond found in hIRF3) constrains the SRD (colored in pink). Residues corresponding to N389–T390–S402 in hIRF3 are all S (S475, S476, and S487) (Supplementary Fig. S5). Phosphorylation of S472 relaxes the contraction of the SRD in IRF7. The majority of the basic-surface forming positively charged amino acids as well as the acidic property of the C-terminal tail were conserved (Supplementary Fig. S5b). Therefore, the roles of IRF7 GVSSL and IRF3 GASSL are interpreted to be consistent.

## Discussion

**Figure 6:**
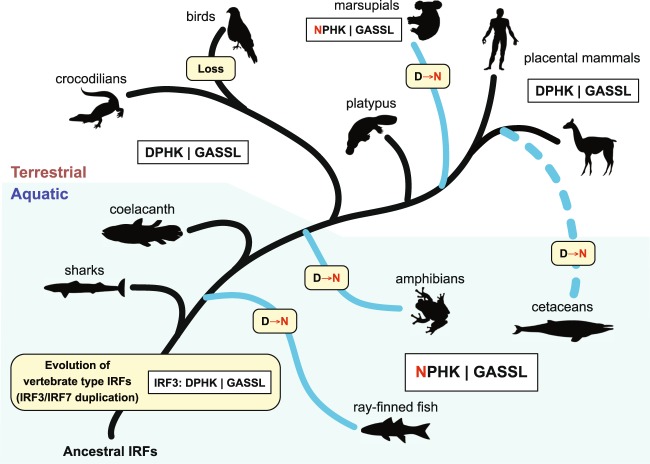
Evolution of the vertebrate IRF3 proteins. The evolution of the two conserved motifs in vertebrate IRF3 proteins is illustrated. The major evolutionary events (duplication, loss, and D → N substitution) are shown in yellow background. The vertebrate lineages that have mainly NPHK in the motif A of IRF3 are shown with solid or dashed blue lines. All animal silhouettes were taken and used without modification from http://www.phylopic.org. Images for sharks, coelacanth, and crocodilians are available under the Public Domain Mark 1.0 license. Images for ray-finned fish, amphibians, birds, marsupials, placental mammals, and cetaceans are available under the Public Domain Dedication 1.0 license. The image of the platypus by Sarah Werning is available under the Creative Commons Attribution 3.0 Unported license (https://creativecommons.org/licenses/by/3.0/).

During the evolution of the IRF protein family, the appearance of the IRF3/7 subfamily coincides with the emergence of jawed vertebrates (Fig. 6). The first vertebrates where the IRF3/7 subfamily has been identified are sharks (cartilaginous fishes), and sharks have a primitive type I IFN system^38,39^. Therefore, the formation of the ancestral-type IRF protein and the duplication that produced IRF3 and 7 proteins must have occurred before the divergence of sharks. It is likely that the IRF3/7 subfamily co-evolved with the emergence of IFNs in the development of vertebrate innate immune systems. Of note is that sharks have developed a unique immune system that may be related to their highly efficient wound healings, and suspected to show a greater resistance to cancers^40,41^.

Our analyses of ~600 IRF protein sequences revealed two uniquely conserved motifs in the IRF3/7 subfamily. They are maintained throughout the evolution of the major vertebrate lineages indicating the functional importance of these motifs in their protein functions. Short motifs and changes in their sequences are known to play significant roles in a wide range of biological and biochemical events, such as signal transduction and glycosylation^42–45^. For the DPHK motif conserved in the IRF3 proteins, the D → N amino acid substitutions to produce the derivative NPHK motif appear to have happened independently at least four times during the vertebrate evolution: in ray-finned fish (after the divergence of reedfish), in amphibians, in marsupials, and in cetaceans (after the divergence of gray whale). Interestingly, three of these four events are associated to lineages that are adapted to aquatic environments (Fig. 6).

Examination of the 3D-structure of the IRF3 DBD revealed the importance of the DPHK motif. In the hIRF3 DBD, the hydrogen-bond network D102–R35–H104 is formed. This hydrogen-bond network is important in determining the orientation of K105, which is one of the DNA contacts (Supplementary Fig. S4). However, in the black carp IRF3 DBD with the D102 → N102 substitution, R35 has been also changed to S35 leading to the loss of the hydrogen-bond network (Fig. 5c). In 57 of the 59 ray-finned fishes we examined, the corresponding position is consistently occupied by either S or C (also one instance of T). Two exceptions are found in the two basal species, reedfish and Dabry’s sturgeon. They have DPHK and closely related EPHK as the motif (Fig. 2a) and both have R35. In the cetacean IRF3s with the NPHK motif, although R35 is still conserved, the hydrogen-bond network has been lost. In marsupial IRF3s, R35 has been changed to hydrophobic amino acids (Y, I, S, and F). Among the five amphibian IRF3s, while R35 is kept in one where DPHK is present (*Microcaecilla unicolor*), in three of the remaining four that have NPHK, R35 has been changed (to S, Q, or C). All other animals including placental mammals, platypus, coelacanth, and sharks, use the R35. All IRF7 proteins also have R in the corresponding position (Supplementary Fig. S4). Therefore, the D → N substitutions in IRF3 must have caused co-evolution in other amino acid positions or *vice versa* to maintain the DNA-binding function. How these changes affected the IRF3 and its DNA-binding functions is yet to be known.

Marine microbes are the dominant life forms in the oceans^46^. The pathogen burdens and the routes of the entries in marine environments are quite different from terrestrial ones. Because the major function of IRF3 is in IFN production, it is thus speculated that the NPHK motif and changes associated with the D → N substitution in IRF3 may facilitate IFN production for aquatic species, specifically through modulations of DNA bindings. In addition, the DPHK (positions 102–105) of human IRF3 is located between nuclear localization (77–89) and nuclear export (139–149) signals^47–50^. Therefore, although the DPHK motif is conserved among vertebrate species and its substitution to NPHK in three groups of animals is all associated with aquatic life, the exact biological functions of the motif need further experimental exploration.

Marsupials are another mammalian species where the NPHK motif is used in IRF3. The newborn marsupials have neither immunological tissues nor organs in their early life. Therefore, the newborns will have to rely totally on their own innate immunity and the passive immunity from their mother to fight off invading pathogens in the non-sterile pouch^51,52^. IRF7 is highly expressed in plasmacytoid dendritic cells and other immune cells and is IFN inducible. IRF7 is likely to be a predominant factor in lymphoid tissue for the production of IFNs. In contrast, IRF3 is ubiquitously expressed and not IFN inducible. Furthermore, IRF3 is known to play a major role in the early stage of IFN productions during viral attacks. It is thus tempting to speculate that IRF3 might play a critical role in newborn’s innate immunity in marsupials. The NPHK motif and the changes associated with the D → N substitution in IRF3 might have facilitated their adaptation for the innate immune responses without the help of IRF7 in newborns.

The GASSL motif is more conserved than the DPHK motif (Fig. 3). The second serine (S386 in hIRF3, Supplementary Fig. S5) is considered the most critical residue for phosphorylation, dimerization, and CBP/p300 binding^36,47,48,53^. Throughout our studies, we have found only one change in S386 in IRF3 and no such change in IRF7 (Supplementary Table S6). The strong selective constraints observed corroborates the experimental evidence indicating the critical role of the S386 in IFN production. The adjacent serine (S385 in hIRF3 and S471 in hIRF7) is also highly conserved and it is changed in only two species of ray-finned fish (Supplementary Table S6). Note that both of them are substitutions to threonine (T), which can also be phosphorylated by serine/threonine kinases. Other serines in SRD may be contributing to fine adjustments of the activations^36,47,48,53^. The glycine (G383 in hIRF3 and G469 in IRF7) is completely conserved in both IRF3 and IRF7 among all the species we examined. Although there is no experimental evidence yet for the significance of the glycine, the strong functional constraint may be explained by the possible involvement of the G383 in capping of the α-helix and stabilization of the loop structure (Fig. 5d–f). It is likely that the G(A/V)SSL motif plays a role in maintaining the signal reception functions of IRF3 and 7.

Aves seem to have only IRF7 and no IRF3^18,30^. Squamates (snakes and lizards), on the other hand, may have compromised IRF7 proteins (Fig. 4b). In a mouse model, IRF7 is the master gene for IFN production^10^. Therefore, one antiviral IRF may be sufficient. Why are two IRFs present in the vast majority of vertebrates during evolution? One possibility is that pathogens activate IFNs through multiple pathways, and all lead to the activation of IRF3 and 7. Hosts have their preferences in sensing various pathogens and activation of IRF3 and 7^15,47,54^. Having the dual factors may have guaranteed the proper responses to various pathogens during evolution. Moreover, aberrant production of IFNs is associated with many types of diseases such as autoimmune disorders^55,56^. The dual factors might offer a tighter regulation of IRF3 and 7 activities in dictating appropriate IFN production for normal IFN-mediated physiological functions. Therefore, the evolutionary adaptation of IRF3 and 7 to various environments might not only play a role in innate immunity, but also other physiological and pathogenic situations.

In summary, other than the hallmark IRF DNA-binding domains, we have identified two additional motifs uniquely conserved in IRF3 and 7. The results obtained in this study suggest that the D → N and associated changes found in the IRF3 DPHK motifs may have played roles in adaptations to aquatic and other environments. Further studies targeted at the functions of the NPHK motif in IRF3 are warranted.

## Materials and Methods

### Searching of IRF3 and IRF7 proteins

The protein sequences of the mouse IRF3 (NP_058545.1) and the mouse IRF7 (NP_058546.1) were used as the queries to perform protein similarity searches using BLASTP^57,58^ against the non-redundant protein database at the National Center for Biotechnology Information (NCBI) with the default options. Sequences were collected from mammals, reptiles, birds, amphibians, and fishes. When more than one isoforms were available from the same species, one isoform that was most similar to those from other species was selected. Protein sequences that were partial and too short were also excluded. In total, IRF3 protein sequences were collected from 62 fish, 125 mammalian, 14 reptilian, and 5 amphibian species. For IRF7, protein sequences were collected from 93 fish, 120 mammalian, 13 reptilian, 50 avian, and 5 amphibian species. All sequences are listed in Supplementary Tables S2–S5.

### Phylogenetic analysis of the representative IRF proteins

To confirm the grouping of IRF proteins, phylogenetic analysis was performed using a set of IRF protein sequences from representative vertebrate species. For each IRF family (from IRF1 to IRF9), protein sequences from the same set of thirteen species were collected (see Supplementary Table S1). The protein sequences were aligned using MAFFT v7.452 with the L-INS-i iterative refinement method^59,60^. The maximum likelihood phylogeny including 116 protein sequences was reconstructed using PhyML v20120412^61^ with the Smart Model Selection using Akaike Information Criterion^62^. Brach support values for the maximum-likelihood phylogeny were calculated using the bootstrap analysis with 500 peudoreplicates. The visualization of the phylogenies was performed using FigTree v1.4.4 (http://tree.bio.ed.ac.uk/software/figtree/).

### Identification of conserved motifs from IRF3 and IRF7 proteins using sequence logos

The representative 25 vertebrate protein sequences from the IRF3/7 subfamily (including 12 IRF3 and 13 IRF7 protein sequences listed in Supplementary Table S1) were aligned as described above. A sequence logo was generated from this alignment using WebLogo v2.8.2^63^ and shown in Supplementary Fig. S2. Excluding the highly conserved N-terminal region where the five tryptophans (Ws) are located, two motifs with at least three continuous conserved amino acids were identified (shown with black dots in Supplementary Fig. S2). To confirm the uniqueness of the two conserved motifs, sequence logos were also generated from alignments containing all IRF subfamilies (116 sequences listed in Supplementary Table S1), each of other IRF subfamilies (1/2, 4/8/9, and 5/6; each including 26 sequences), as well as IRF3 and IRF7 protein sequences separately (66 sequences each used for phylogenetic analysis below).

### Phylogenetic analysis of the IRF3 and IRF7 proteins

Phylogenetic analyses were performed for larger datasets of IRF3 and IRF7 protein sequences. For each of IRF3 and IRF7, three datasets including 46 species of fishes, 65 species of mammals, reptiles, and amphibians, as well as 40 species of birds were generated (see Supplementary Tables S2–S5). For the second dataset including mammals, reptiles, and amphibians, a coelacanth sequence was also included as the outgroup. Multiple sequence alignment and phylogenetic analysis were done for each dataset as described above. All datasets including multiple sequence alignments are available upon request.

### Structural modeling of IRF3 and IRF7 proteins

For the human IRF3 protein (UniProt: Q14653, including DPHK and GASSL motifs), the 3D-structures were solved for the N-terminal DBD region (PDB 2O6G; positions 1–112 aa) and for the C-terminal region including IAD and SRD (PDB 1QWT; positions 190–422 aa) separately^33,34^. For the linker region (positions 113–189 aa), 3D-structure has not been reported yet.

The structure of the IRF3 DBD with the NPHK motif found in the black carp (*Mylopharyngodon piceus*, NCBI: QAT77258.1) was modeled using the human IRF3 DBD structure (PDB 2PI0) as the template and using the SWISS-MODEL server^64^. The QMEAN score of the model^65^ was −1.77. The root mean square distance of the main chain (RMSD) for the model was 0.45 Å. QMEAN > − 4 and RMSD < 1 Å are considered to be acceptable ranges. The model was therefore considered usable. The structure of the C-terminal domain of the human IRF7 with the GVSSL motif (Q92985) was modeled using the structure of the human IRF3 C-terminal region (PDB: 1QWT) as the template. The modeled structure had values for QMEAN and RMSD as −3.40 and 0.22 Å, respectively, again in the acceptable range. Structural mining and preparation of graphics were performed using the PyMOL Molecular Graphics System, version 2.3.2 (Schrödinger, LLC, New York, NY, USA).

## Supplementary information

Supplementary information.
