# Supplementary material for: Adaptations of Interferon Regulatory Factor 3 with Transition from Terrestrial to Aquatic Life
Source: Sci Rep. 2020 Mar 11;10:4508. doi: 10.1038/s41598-020-61365-9 (PMC7066157; doi:10.1038/s41598-020-61365-9)
Supplement: Supplementary file 1 — Supplementary information. [file 41598_2020_61365_MOESM1_ESM.pdf]

Supplementary Information

Adaptations of Interferon Regulatory Factor 3 with Transition from Terrestrial to Aquatic Life

Monica Angeletti[1], Wan-Ling Nicole Hsu[1#], Nashaat Majo[1], Hideaki Moriyama[1], Etsuko N. Moriyama[1,2] *, and Luwen Zhang[1,3]*

School of Biological Sciences[1], Center for Plant Science Innovation[2,] Nebraska Center for Virology[3], University of Nebraska, Lincoln, NE 68583

*Corresponding author

Table S1: Protein sequences used for each IRF family.

Species	IRF1	IRF2	IRF3	IRF4	IRF5	IRF6	IRF7	IRF8	IRF9
**[Lobe-finned fish]**									
Latimeria chalumnae (West Indian Ocean coelacanth)	XP_014350775.1	XP_014351568.1	XP_005991012.1	XP_005995158.1	XP_006010437.1	XP_014347913.1	XP_005995343.1	XP_006010434.1	XP_006010095.1[2]
**[Amphibia]**									
Xenopus laevis (African clawed frog)	NP_001085588.1	NP_001088726.1	NP_001079588.1	XP_018124943.1	XP_018106598.1	NP_001081215.1	XP_018113086.1	NP_001087097.1	NP_001084846.1
**[Reptiles and birds]**									
Alligator mississippiensis (American alligator)	XP_006261200.2	XP_006263935.1	XP_006274938.1	XP_006276607.1	XP_006261193.2	KYO27854.1	XP_014459685.1	XP_006276758.1	KYO24165.1
Gallus gallus (Red junglefowl, chicken)	AAA62160.1	NP_990527.1		NP_989630.1	NP_001026758.1	XP_015154525.1	NP_990703.1[1]	NP_990747.1	NP_989889.1
**[Mammals: monotrema]**									
Ornithorhynchus anatinus (Platypus)	XP_028907840.1	XP_028932783.1	XP_028929100.1	XP_028909458.1	XP_028929175.1	XP_001508806.1	XP_028915201.1	XP_001507292.1	XP_028934200.1
**[Mammals: marsupials]**									
Vombatus ursinus (Wombat)	XP_027704973.1	XP_027693199.1	XP_027715067.1	XP_027697513.1	XP_027695980.1	XP_027726959.1	XP_027716012.1	XP_027703450.1	XP_027698833.1
**[Mammals: placentals]**									
Zalophus californianus (California sea lion)	XP_027463006.1	XP_027453865.1	XP_027475317.1	XP_027459737.1	XP_027429439.1	XP_027469234.1	XP_027436269.1	XP_027474849.1	XP_027428451.1
Sus scrofa (Pig)	NP_001090882.1	XP_005671760.1	NP_998935.1	XP_020953623.1	XP_013846259.1	NP_999443.1	NP_001090897.1	NP_001239356.1	NP_001072138.1
Bos taurus (Cattle)	NP_001178190.1	NP_001192722.1	NP_001025016.1	NP_001193091.1	NP_001030542.1	NP_001070402.1	XP_024842714.1	NP_001077238.1	XP_010807205.1
Rattus norvegicus (Brown rat)	NP_036723.1	ABG67970.1	NP_001006970.1	NP_001099578.1	NP_001100056.1	NP_001102329.1	NP_001028863.1	NP_001008722.1	AAH12968.1
Mus musculus (House mouse)	NP_001152868.1	NP_032417.3	NP_058545.1	NP_038702.1	NP_036187.1	NP_058547.2	NP_058546.1	NP_032346.1	AAH79454.1
Macaca mulatta (Rhesus macaque)	XP_001104294.1	NP_001129265.1	NP_001129269.1	XP_001118967.2	XP_001092569.1	XP_001110321.1	XP_014968666.2	XP_001084409.1	NP_001247598.1
Homo sapiens (Human)	NP_002189.1	NP_002190.2	NP_001562.1	NP_002451.2	NP_001092099.1	AEL89176.1	AAC70999.1	EAW95435.1	NP_006075.3

[1]Although the entry is annotated as IRF3, it clusters with IRF7 (see Figure S1).
[2]Although the entry is annotated as "IRF8 like", it clusters with IRF9 (see Figure S1).

Table S2: Protein sequences used for mammalian IRF3 and IRF7.[1]

Species[2]	Common name	Order	IRF3 Acc #	IRF7 Acc #	IRF3 motifs		IRF7 motifs	
					A[3]	B[4]	A[3]	B[4]
**[Monotremata]**								
*Ornithorhynchus anatinus**	Platypus	Monotremata	XP_028929100.1	XP_028915201.1	DPHK	GASSL	DPHK	GASSL
**[Marsupials]**								
*Sarcophilus harrisii**	Tasmanian devil	Dasyuromorphia	XP_023355902.1	XP_023362391.1	NPHK	GASSL	DPHK	GVSSL
*Monodelphis domestica**	Opossum	Didelphimorphia	XP_001379797.2		KPHK	GASSL		
*Phascolarctos cinereus**	Koala	Diprotodontia	XP_020859305.1	XP_020835087.1	NPHK	GASSL	DPHK	GVSSL
*Vombatus ursinus**	Wombat	Diprotodontia	XP_027715067.1	XP_027716012.1	NPHK	GASSL	DPHK	GISSL
**[Placental mammals: Laurasiatheria]**								
Erinaceus europaeus	European hedgehog	Eulipotyphla	XP_016047279.1		DPHK	GASSL		
*Sorex araneus**	Common shrew	Eulipotyphla	XP_012790744.1	XP_004603181.1	DPHK	GASSL	DPHK	GASSL
Condylura cristata	Star-nosed mole	Eulipotyphla	XP_004694067.1		DPHK	GASSL		
*Manis javanica**	Pangolin	Pholidota	XP_017521112.1	XP_017534309.1	DPHK	GASSL	DPHK	GASSL
Acinonyx jubatus	Cheetah	Carnivora	XP_026893938.1	XP_026907208.1	DPHK	GASSL	DPHK	GVSSL
Puma concolor	Cougar	Carnivora	XP_025770394.1		DPHK	GASSL		
*Felis catus**	Domestic cat	Carnivora	XP_003997552.3	XP_011285475.1	DPHK	GASSL	DPHK	GVSSL
Panthera pardus	Leopard	Carnivora	XP_019281194.1	XP_019293176.1	DPHK	GASSL	DPHK	GVSSL
Panthera tigris altaica	Tiger	Carnivora	XP_007074239.1		DPHK	GASSL		
Canis lupus dingo	Dingo	Carnivora	XP_025280023.1	XP_025308435.1	DPHK	GASSL	DPHK	GVSSL
Lynx pardinus	Iberian lynx	Carnivora		VFV36246.1			DPHK	GVSSL
Puma concolor	Cougar	Carnivora		XP_025771441.1			DPHK	GVSSL
*Vulpes vulpes**	Red fox	Carnivora	XP_025869690.1	XP_025847483.1	DPHK	GASSL	DPHK	GVSSL
Neomonachus schauinslandi	Monk seal	Carnivora	XP_021536967.1	XP_021547835.1	DPHK	GASSL	DPHK	GVSSL
Leptonychotes weddellii	Seal	Carnivora	XP_006749345.1	XP_006741842.1	DPHK	GASSL	DPHK	GVSSL
Odobenus rosmarus divergens	Walrus	Carnivora	XP_004409946.1	XP_004403823.1	DPHK	GASSL	DPHK	GVSSL
Callorhinus ursinus	Northern fur seal	Carnivora	XP_025705268.1	XP_025717869.1	DPHK	GASSL	DPHK	GVSSL
*Zalophus californianus**	California sea lion	Carnivora	XP_027475317.1	XP_027436269.1	DPHK	GASSL	DPHK	GVSSL
Eumetopias jubatus	Steller sea lion	Carnivora	XP_027947261.1	XP_027950114.1	DPHK	GASSL	DPHK	GVSSL
*Mustela putorius furo**	Ferret	Carnivora	XP_012919390.1	XP_004781195.2	DPHK	GASSL	DPHK	GVSSL
Enhydra lutris kenyoni	Sea otter	Carnivora	XP_022379584.1	XP_022363083.1	DPHK	GASSL	DPHK	GVSSL
Ailuropoda melanoleuca	Giant panda	Carnivora	XP_002917881.1	XP_019665733.1	DPHK	GASSL	DPHK	GVSSL
Ursus arctos horribilis	Grizzly bear	Carnivora	XP_026337601.1	XP_026346160.1	DPHK	GASSL	DPHK	GVSSL
Ursus maritimus	Polar bear	Carnivora	XP_008682776.1	XP_008697887.1	DPHK	GASSL	DPHK	GVSSL
*Pteropus alecto**	Black flying fox	Chiroptera	XP_006905084.1	XP_024901895.1	DPHK	GASSL	DPHK	GVSSL
Pteropus vampyrus	Large flying fox	Chiroptera	XP_011372830.1	XP_011379114.1	DPHK	GASSL	DPHK	GVSSL

Rousettus aegyptiacus	Egyptian rousette	Chiroptera	XP_015977865.1	XP_015993660.1	DPHK	GASSL	DPHK	GVSSL
*Hipposideros armiger**	Great roundleaf bat	Chiroptera	XP_019513839.1	XP_019486625.1	DPHK	GASSL	DPHK	GVSSL
*Desmodus rotundus**	Common vampire bat	Chiroptera	XP_024422099.1	XP_024430322.1	DPHK	GASSL	DPHK	GASSL
Phyllostomus discolor	Pale spear-nosed bat	Chiroptera	XP_028385987.1	XP_028370927.1	DPHK	GASSL	DPHK	GVSSL
Miniopterus natalensis	Natal long-fingered bat	Chiroptera	XP_016061535.1	XP_016075093.1	DPHK	GASSL	DPHK	GASSL
Eptesicus fuscus	Brown bat	Chiroptera	XP_008152570.1	XP_027992302.1	DPHK	GASSL	DPHK	GVSSL
*Myotis davidii**	David's myotis	Chiroptera	XP_015420914.1	AOQ26356.1	DPHK	GASSL	DPHK	GASSL
Myotis brandtii	Brandt's bat	Chiroptera	XP_005879595.1		DPHK	GASSL		
Myotis lucifugus	Little brown bat	Chiroptera	XP_006107879.1		DPHK	GASSL		
*Ceratotherium simum simum**	White rhinoceros	Perissodactyla	XP_004440345.1	XP_004441106.1	DPHK	GASS**M**	DPHK	GVSSL
Equus asinus	Donkey	Perissodactyla	XP_014685801.1	XP_014687003.1	DPHK	GASS**M**	DPHK	GVSSL
*Equus caballus**	Horse	Perissodactyla	XP_014585307.1	XP_023510517.1	DPHK	GASS**M**	DPHK	GVSSL
Equus przewalskii	Przewalski's horse	Perissodactyla	XP_008506849.1		DPHK	GASS**M**		
*Vicugna pacos**	Alpaca	Artiodactyla	XP_006208513.2	XP_015107472.1	DPHK	GASSL	DPHK	GVSSL
Camelus dromedarius	Arabian camel	Artiodactyla	XP_010991480.1	TKS39661.1	DPHK	GASSL	DPHK	GVSSL
Camelus bactrianus	Camel	Artiodactyla	XP_010945290.1	XP_010954447.1	DPHK	GASSL	DPHK	GVSSL
Camelus ferus	Camel	Artiodactyla	XP_006173854.1		DPHK	GASSL		
*Sus scrofa**	Pig	Artiodactyla	NP_998935.1	NP_001090897.1	DPHK	GASSL	DPHK	GVSSL
*Odocoileus virginianus texanus**	White tailed deer	Artiodactyla	XP_020741254.1	XP_020767809.1	DPHK	GASSL	DPHK	GVSSL
Cervus elaphus hippelaphus	Central European red deer	Artiodactyla		OWK10783.1			DPHK	–
Bubalus bubalis	Water buffalo	Artiodactyla	XP_025125790.1	XP_006045842.1	DPHK	GASSL	DPHK	GVSSL
Bos mutus	Wild yak	Artiodactyla	XP_005905061.1	XP_005895176.1	DPHK	GASSL	DPHK	GVSSL
*Bos taurus**	Cattle	Artiodactyla	NP_001025016.1	XP_024842714.1	DPHK	GASSL	DPHK	GVSSL
Bos indicus x Bos taurus	Hybrid cattle	Artiodactyla		XP_027388749.1			DPHK	GVSSL
Bison bison bison	Bison	Artiodactyla	XP_010848014.1	XP_010834331.1	DPHK	GASSL	DPHK	GVSSL
Ovis aries	Sheep	Artiodactyla	XP_004015427.2	XP_027815763.1	DPHK	GASSL	DPHK	GVSSL
Ovis aries musimon	Mouflon	Artiodactyla		XP_012023997.1			DPHK	GVSSL
Pantholops hodgsonii	Tibetan antelope	Artiodactyla	XP_005955456.1	XP_005981955.1	DPHK	GASSL	DPHK	GVSSL
*Capra hircus**	Goat	Artiodactyla	XP_013826927.2	XP_017898523.1	DPHK	GASSL	DPHK	GVSSL
Balaenoptera acutorostrata scammoni	Minke whale	Cetacea	XP_007185012.1		DPHK	GASSL		
*Eschrichtius robustus**	Gray whale	Cetacea	MBV98132.1	MBW00388.1	DPHK	GASSL	DPHK	–
*Physeter catodon**	Sperm whale	Cetacea	XP_007112499.1	XP_028341711.1	NPHK	GASSL	DPHK	GASSL
*Lipotes vexillifer**	Baiji	Cetacea	XP_007463434.1	XP_007465980.1	NPHK	GASSL	DPHK	GASSL
Lagenorhynchus obliquidens	Pacific white-sided dolphin	Cetacea	XP_026936043.1	XP_026946429.1	NPHK	GASSL	DPHK	GASSL
*Orcinus orca**	Killer whale	Cetacea	XP_004286221.1	XP_012391568.1	NPHK	GASSL	DPHK	GASSL
Sousa chinensis	Indian Ocean humpback dolphin	Cetacea	TEA25515.1		NPHK	GASSL		
Monodon monoceros	Narwhal	Cetacea	XP_029063320.1	XP_029062954.1	NPHK	GASSL	DPHK	GASSL

Neophocaena asiaeorientalis asiaeorientalis	Finless porpoise	Cetacea	XP_024624254.1	XP_024605042.1	NPHK	GASSL	DPHK	GASSL
*Delphinapterus leucas**	Beluga whale	Cetacea	XP_022440768.1	XP_022420974.1	NPHK	GASSL	DPHK	GASSL
**[Placental mammals: Euarchontoglires]**								
*Marmota marmota marmota**	Alpine marmot	Rodentia	XP_015352013.1	XP_015358869.1	DPHK	GASSL	DPHK	GVSSL
Marmota monax	Groundhog	Rodentia	ACC60989.1		DPHK	GASPL		
Marmota flaviventris	Yellow-bellied marmot	Rodentia		XP_027809360.1			DPHK	GVSSL
Urocitellus parryii	Ground squirrel	Rodentia	XP_026262098.1	XP_026235122.1	DPHK	GASSL	DPHK	GVSSL
Ictidomys tridecemlineatus	Ground squirrel	Rodentia	XP_021589862.1	XP_013221293.1	DPHK	GASSL	DPHK	GVSSL
*Castor canadensis**	American beaver	Rodentia	XP_020015557.1	XP_020037622.1	DPHK	GASSL	DPHK	GVSSL
Dipodomys ordii	Kangaroo rat	Rodentia	XP_012883150.1	XP_012888821.1	DPHK	GASSL	DPHK	GVSSL
*Jaculus jaculus**	Lesser Egyptian jerboa	Rodentia	XP_004672342.1	XP_004653953.1	DPHK	GASSL	DPHK	GVSSL
*Nannospalax galili**	Blind mole-rat	Rodentia	XP_008843304.1	XP_008852618.1	DPHK	GASSL	DPHK	GVSSL
*Mesocricetus auratus**	Golden hamster	Rodentia	XP_012979714.1	XP_005063402.1	DPHK	GASSL	DPHK	GVSSL
Cricetulus griseus	Chinese hamster	Rodentia	XP_027276506.1	XP_003509823.1	DPHK	GASSL	DPHK	GVSSL
Microtus ochrogaster	Prairie vole	Rodentia	XP_005366888.2	XP_005351563.1	DPHK	GASSL	DPHK	GVSSL
Neotoma lepida	Desert woodrat	Rodentia	OBS74478.1	OBS70876.1	DPHK	GASSL	DPHK	GVSSL
*Peromyscus maniculatus bairdii**	Deer mouse	Rodentia	XP_015860325.1	XP_015851547.1	DPHK	GASSL	DPHK	GVSSL
Peromyscus leucopus	White-footed mouse	Rodentia	XP_028717692.1	XP_028725932.1	DPHK	GASSL	DPHK	GVSSL
Meriones unguiculatus	Mongolian gerbil	Rodentia	XP_021489617.1	XP_021501900.1	DPHK	GASSL	DPHK	GASSL
Grammomys surdaster	Arid thicket rat	Rodentia	XP_028644756.1	XP_028642672.1	DPHK	GASSL	DPHK	GASSL
*Rattus norvegicus**	Brown rat	Rodentia	NP_001006970.1	NP_001028863.1	DPHK	GASSL	DPHK	GVSSL
Mus pahari	Gairdner's shrewmouse	Rodentia	XP_021069816.1	XP_021070709.1	DPHK	GASSL	DPHK	GASSL
Mus caroli	Ryukyu mouse	Rodentia	XP_021023153.1	XP_021022685.1	DPHK	GASSL	DPHK	GVSSL
*Mus musculus**	House mouse	Rodentia	NP_058545.1	NP_058546.1	DPHK	GASSL	DPHK	GVSSL
Fukomys damarensis	Damara mole-rat	Rodentia	XP_010639014.1	XP_010638643.1	DPHK	GASSL	DPHK	GTSSL
*Heterocephalus glaber**	Naked mole-rat	Rodentia	XP_012921780.1	EHB16346.1	DPHK	GASSL	DPHK	GTSSL
*Cavia porcellus**	Guinea pig	Rodentia	XP_003465554.1	XP_004999585.1	DPHK	GASSL	DPHK	GTSSL
*Chinchilla lanigera**	Long-tailed chinchilla	Rodentia	XP_005412915.1	XP_005401881.1	DPHK	GASSL	DPHK	GTSSL
Octodon degus	Common degu	Rodentia	XP_012368202.1	XP_023578534.1	DPHK	GASSL	DPHK	GTSSL
*Oryctolagus cuniculus**	European rabbit	Lagomorpha	XP_008251035.1	XP_008251920.1	DPHK	GASSL	DPHK	GVSSL
Ochotona princeps	American pika	Lagomorpha	XP_004597221.1	XP_012786419.1	DPHK	GASSL	DPHK	GLSSL
*Tupaia chinensis**	Chinese tree shrew	Scandentia	ELW47132.1	XP_006164496.1	DPHK	GASSL	DPHK	GVSSL
*Galeopterus variegatus**	Flying lemur	Dermoptera	XP_008573613.1	XP_008592045.1	DPHK	GASSL	DPHK	GASSL
*Otolemur garnettii**	Galago	Primates	XP_003801591.2	XP_003802849.1	DPHK	GASSL	DPHK	GVSSL
Propithecus coquereli	Sifaka	Primates	XP_012498623.1	XP_012507502.1	DPHK	GASSL	DPHK	GVSSL
*Microcebus murinus**	Mouse lemur	Primates	XP_012609835.1	XP_012624944.1	DPHK	GASSL	DPHK	GVSSL
Carlito syrichta	Tarsier	Primates	XP_008046593.1		DPHK	GASSL		

*Saimiri boliviensis boliviensis**	Squirrel monkey	Primates	XP_010349389.1	XP_003943408.1	DPHK	GASSL	DPHK	GVSSL
*Callithrix jacchus**	Common marmoset	Primates	XP_008986666.1	XP_002755734.1	DPHK	GASSL	DPHK	GVSSL
Cebus capucinus imitator	Panamanian white-faced capuchin	Primates	XP_017376552.1	XP_017363356.1	DPHK	GASSL	DPHK	GVSSL
Aotus nancymaae	Nancy Ma's night monkey	Primates	XP_012316297.1	XP_012311512.1	DPHK	GASSL	DPHK	GVSSL
*Piliocolobus tephrosceles**	Ugandan red colobus	Primates	XP_023038071.1	XP_023039216.1	DPHK	GASSL	DPHK	GVSSL
Colobus angolensis palliatus	Angola colobus	Primates	XP_011801751.1	XP_011785390.1	DPHK	GASSL	DPHK	GVSSL
Rhinopithecus roxellana	Golden snub-nosed monkey	Primates	XP_010367793.1	XP_010350831.1	DPHK	GASSL	DPHK	GVSSL
Rhinopithecus bieti	Black snub-nosed monkey	Primates	XP_017737655.1	XP_017749500.1	DPHK	GASSL	DPHK	GVSSL
Chlorocebus sabaeus	Green monkey	Primates	XP_007995777.1	XP_007979751.1	DPHK	GASSL	DPHK	GVSSL
Mandrillus leucophaeus	Drill	Primates	XP_011821415.1	XP_011849212.1	DPHK	GASSL	DPHK	GVSSL
Papio anubis	Olive baboon	Primates	XP_009193252.1	XP_021781059.1	DPHK	GASSL	DPHK	GVSSL
Theropithecus gelada	Gelada	Primates	XP_025223664.1	XP_025212542.1	DPHK	GASSL	DPHK	GVSSL
Cercocebus atys	Sooty mangabey	Primates	XP_011936286.1	XP_011896391.1	DPHK	GASSL	DPHK	GVSSL
*Macaca mulatta**	Rhesus macaque	Primates	NP_001129269.1	XP_014968666.2	DPHK	GASSL	DPHK	GVSSL
Macaca fascicularis	Crab-eating macaque	Primates	XP_005589990.1	XP_005576827.1	DPHK	GASSL	DPHK	GVSSL
Macaca nemestrina	Southern pig-tailed macaque	Primates		XP_011760597.1			DPHK	GVSSL
*Pongo abelii**	Sumatran orangutan	Primates	XP_009231179.1	XP_009244254.1	DPHK	GASSL	DPHK	GVSSL
*Homo sapiens**	Human	Primates	NP_001562.1	AAC70999.1	DPHK	GASSL	DPHK	GVSSL
Gorilla gorilla gorilla	Gorilla	Primates	XP_018870991.1	XP_018892692.1	DPHK	GASSL	DPHK	GVSSL
Pan paniscus	Bonobo	Primates	XP_003814385.1	XP_008975721.1	DPHK	GASSL	DPHK	GVSSL
Pan troglodytes	Chimpanzee	Primates	XP_016792049.1	XP_016775486.1	DPHK	GASSL	DPHK	GVSSL
Nomascus leucogenys	Northern white-cheeked gibbon	Primates	XP_012365342.1	XP_012359243.1	DPHK	GASSL	DPHK	GVSSW
**[Placental mammals: Xenarthra]**								
*Dasypus novemcinctus**	Nine-banded armadillo	Cingulata	XP_023438602.1	XP_004459829.1	DPHK	GASSL	DPHK	GASSL
**[Placental mammals: Afrotheria]**								
*Trichechus manatus latirostris**	Florida manatee	Sirenia	XP_004381699.1	XP_004389466.1	DPHK	GASSL	DPHK	GTSSL
*Orycteropus afer afer**	Aardvark	Tubulidentata	XP_007941039.1	XP_007947551.1	DPHK	GASSL	DPHK	GISSL
Loxodonta africana	African bush elephant	Proboscidea		XP_023398114.1			DPHK	GTSSL
*Echinops telfairi**	Lesser hedgehog tenrec	Afrosoricida	XP_004710496.1	XP_004717251.1	DPHK	GASSL	DPHK	GASSL
Chrysochloris asiatica	Cape golden mole	Afrosoricida	XP_006868475.1	XP_006877067.1	DPHK	GASSL	DPHK	GASSL
*Elephantulus edwardii**	Cape elephant shrew	Macroscelidea	XP_006898787.1	XP_006893405.1	DPHK	GASSL	DPHK	GASSL

[1]Total numbers of sequences used are 125 for IRF3 and 120 for IRF7.
[2]Fifty-four species included in the phylogenetic analysis (Figure 4) are marked with *.
[3]Motif A color code used: DPHK in red, NPHK in blue, and all amino acid substitutions found are shown in black letters.
[4]Motif B color code used: GASSL in green, GVSSL in blue, and all amino acid substitutions found are shown in black letters. '-' indicates the motif sequence was not identified (including also a possible incomplete sequence).

Table S3: Protein sequences used for reptilian and amphibian IRF3 and IRF7.[1]

Species[2]	Common name	Order	IRF3 Acc #	IRF7 Acc #	IRF3 motifs		IRF7 motifs	
					A[3]	B[4]	A[3]	B[4]
**[Reptiles]**								
*Alligator mississippiensis**	American alligator	Crocodilia	XP_006274938.1	XP_014459685.1	DPHK	GASSL	DPHK	GTSSL
Alligator sinensis	Chinese alligator	Crocodilia	XP_025051908.1	XP_006019280.1	DPHK	GASSL	DPHK	GTSSL
Gavialis gangeticus	Gharial	Crocodilia		XP_019375349.1			DPHK	GTSSL
Crocodylus porosus	Saltwater crocodile	Crocodilia		XP_019402849.1			DPHK	GTSSL
*Terrapene carolina triunguis**	Three-toed box turtle	Testudines	XP_026502235.1	XP_026513417.1	DPHK	GASSL	DPHK	GASSL
Chrysemys picta bellii	Painted turtle	Testudines	XP_005280819.1	XP_005290671.1	DPHK	GASSL	DPHK	GASSL
Chelonia mydas	Green sea turtle	Testudines		XP_027679112.1			DPHK	GASSL
Pelodiscus sinensis	Chinese softshell turtle	Testudines		XP_006124947.2			DPHK	GASSL
*Gekko japonicus**	Schlegel's Japanese gecko	Squamata	XP_015263994.1		DPHK	GASSL		
*Anolis carolinensis**	Green anole	Squamata	XP_003222748.2	XP_008106753.1	DPHK	GASSL	DYH	GVSSL
*Podarcis muralis**	Common wall lizard	Squamata	XP_028558674.1	XP_028591258.1	DPHK	GASSL	DYH	GFSSL
*Pogona vitticeps**	Central bearded dragon	Squamata	XP_020649690.1	XP_020639822.1	DPHK	GASSL	DFH	GASSL
Python bivittatus	Burmese python	Squamata	XP_015742861.1		DPHK	GASSL		
Protobothrops mucrosquamatus	Brown spotted pit viper	Squamata	XP_015665683.1		DPHK	GASSL		
Thamnophis sirtalis	Common garter snake	Squamata	XP_013927360.1		DPHK	GASSL		
Ophiophagus hannah	King cobra	Squamata	ETE70933.1		DPHK	GASSL		
*Pseudonaja textilis**	Eastern brown snake	Squamata	XP_026566600.1	XP_026562421.1	DPHK	GASSL	DFR	GCSSL
Notechis scutatus	Tiger snake	Squamata	XP_026525424.1	XP_026523996.1	DPHK	GASSL	DFR	GCSSL
**[Amphibians]**								
*Rhinatrema bivittatum**	Two-lined caecilia	Apoda	XP_029441215.1	XP_029438614.1	HPHK	GASSL	DPHK	GASSL
*Microcaecilia unicolor**	Tiny Cayenne caecilia	Apoda	XP_030053868.1	XP_030057150.1	DPHK	GASSL	DPHK	GASSL
*Nanorana parkeri**	Mountain slow frog	Anura	XP_018424622.1	XP_018411535.1	NPHK	GASSL	DPHK	GASSL
Xenopus tropicalis	Western clawed frog	Anura	XP_017951335.1	XP_012817837.1	NPHK	GASSL	DPHK	GASSI
*Xenopus laevis**	African clawed frog	Anura	NP_001079588.1	XP_018113086.1	NPHK	GASSL	DPHK	GASSL

[1]Total numbers of sequences used are 19 for IRF3 and 18 for IRF7.
[2]Eleven species included in the phylogenetic analysis (Figure 4) are marked with *.
[3]Motif A color code used: DPHK in red, NPHK in blue, and all amino acid substitutions found are shown in black letters.
[4]Motif B color code used: GASSL in green, GVSSL in blue, and all amino acid substitutions found are shown in black letters.

Table S4: Protein sequences used for fish IRF3 and IRF7.[1]

Species[2]	Common name	Order	IRF3 Acc #	IRF7 Acc #	IRF3 motifs A[3]	IRF3 motifs B[4]	IRF7 motifs A[3]	IRF7 motifs B[4]
**[Cartilaginous fish]**								
*Callorhinchus milii**	Australian ghostshark	Chimaeriformes	XP_007884262.1	XP_007907546.1	DPHK	GASSL	DPHK	GASSL
*Chiloscyllium griseum**	Grey bamboo shark	Orectolobiformes	CDO19214.1	CDO19215.1	RPHL	GASSL	DPHK	GASSL
**[Lobe-finned fish]**								
*Latimeria chalumnae**	West Indian Ocean coelacanth	Coelacanthiformes	XP_005991012.1	XP_005995343.1	DPHK	GASSL	DPHK	GASSL
**[Ray-finned fish]**								
*Erpetoichthys calabaricus**	Reedfish	Polypteriformes	XP_028669438.1	XP_028649350.1	DPHK	GASSL	DPHK	GASSL
*Acipenser dabryanus**	Dabry's sturgeon	Acipenseriformes	AUD39943.1		EPHK	GASSL		
*Acipenser ruthenus**	Sterlet	Acipenseriformes		RXM32035.1			DPHK	GASSL
*Lepisosteus oculatus**	Spotted gar	Lepisosteiformes	XP_015208825.1	XP_015193511.1	NPHK	GASSL	DPHK	GATSI
*Anguilla anguilla**	European eel	Anguilliformes	AHA98254.1	AHA98255.1	-	GASSL	DPHK	GASSL
*Paramormyrops kingsleyae**	Elephantfish	Osteoglossiformes	XP_023681797.1	XP_023697785.1	NPHK	GASSL	DPHK	GASSL
*Scleropages formosus**	Asian arowana	Osteoglossiformes	XP_018592422.1	KPP75092.1	NPHK	GASSL	DPHK	GASSL
*Denticeps clupeoides**	Denticle herring	Clupeiformes	XP_028824534.1	XP_028814017.1	NPHK	GASSL	DPHK	GASSI
*Clupea harengus**	Atlantic herring	Clupeiformes	XP_012672802.1	XP_012692802.1	NPHK	GASSL	NPHK	GASSL
*Pangasianodon hypophthalmus**	Iridescent shark	Siluriformes	XP_026770382.1	XP_026774500.1	NPHK	GASSL	NPHK	GASSL
Ictalurus punctatus	Channel catfish	Siluriformes	AHH37201.1	XP_017341270.1	NPHK	GATSL	NPHK	GASSL
Tachysurus fulvidraco	Yellowhead catfish	Siluriformes		XP_027028906.1			NPHK	GASSL
*Electrophorus electricus**	Electric eel	Gymnotiformes	XP_026886962.1	XP_026872213.1	NPHK	GASSL	NPHK	GASSL
*Danio rerio**	Zebrafish	Cypriniformes	XP_017213957.1	NP_956971.2	DPHK	GASSL	DQHK	GASSL
*Mylopharyngodon piceus**	Black carp	Cypriniformes	QAT77258.1	AVX27623.1	NPHK	GASSL	DQHK	GASSL
Squaliobarbus curriculus	Barbel chum	Cypriniformes	AQV11930.1	AMP81961.1	NPHK	GASSR	DQHK	GASSL
Ctenopharyngodon idella	Grass carp	Cypriniformes		ACS34986.1			DQHK	GASSL
Gobiocypris rarus		Cypriniformes		AWR88268.1			DQHK	GASSL
*Carassius auratus**	Goldfish	Cypriniformes	XP_026133080.1	XP_026095177.1	NPHK	GASSL	DQHK	GASSL
*Cyprinus carpio**	Common carp	Cypriniformes	XP_018957527.1	ADZ55457.1	NPHK	GASSL	DQHK	GASSL
Sinocyclocheilus grahami	Golden-line barbell	Cypriniformes		XP_016114969.1			DQHK	GASSL
*Schizopygopsis pylzovi**		Cypriniformes	AUL77374.1	AUL77424.1	NPHK	GASSL	DQHK	GASSL
Schizopygopsis stoliczkai	False osman	Cypriniformes		AUL77421.1			DQHK	GASSL
Ptychobarbus dipogon		Cypriniformes		AUL77431.1			DQHK	GASSL
Gymnocypris eckloni		Cypriniformes	AUL77372.1	AUL77422.1	NPHK	GASSL	DQHK	GASSL
Platypharodon extremus		Cypriniformes		AUL77425.1			DQHK	GASSL

Gymnocypris przewalskii		Cypriniformes		AMB19592.1			DQHK	GASSL
Oxygymnocypris stewartii		Cypriniformes		AUL77426.1			DQHK	GASSL
Gymnocypris waddellii		Cypriniformes		AUL77420.1			DQHK	GASSL
Gymnodiptychus pachycheilus		Cypriniformes		AUL77423.1			DQHK	GASSL
Schizothorax macropogon		Cypriniformes		AUL77427.1			DQHK	GASSL
Schizothorax labiatus	Kunar snowtrout	Cypriniformes		AUL77432.1			DQHK	GASSL
Schizothorax lissolabiata		Cypriniformes		AUL77419.1			DQHK	GASSL
Schizothorax lantsangensis		Cypriniformes		AUL77428.1			DQHK	GASSL
*Esox lucius**	Northern pike	Esociformes	XP_010895875.1	XP_010881979.1	NPHK	GASSL	DPHK	GASSL
*Salvelinus alpinus**	Arctic char	Salmoniformes	XP_023863977.1	XP_023826405.1	NPNK	GASSL	DPHK	GASSL
*Oncorhynchus nerka**	Sockeye salmon	Salmoniformes	XP_029485636.1	XP_029490329.1	NPNK	GASSL	DPHK	GASSL
Oncorhynchus kisutch	Coho salmon	Salmoniformes		XP_020336522.1			DPHK	GASSL
Oncorhynchus tshawytscha	Chinook salmon	Salmoniformes		XP_024268976.1			DPHK	GASSL
Oncorhynchus mykiss	Rainbow trout	Salmoniformes	NP_001244191.1	XP_021456174.1	NPNK	GASSP	DPHK	GASSL
Salmo trutta	Brown trout	Salmoniformes		XP_029552514.1			DPHK	GASSL
Salmo salar	Atlantic salmon	Salmoniformes		NP_001130020.1			DPHK	GASSL
Gouania willdenowi	Blunt-snouted clingfish	Gobiesociformes	XP_028331811.1		NPHK	GASSL		
*Kryptolebias marmoratus**	Mangrove rivulus	Cyprinodontiformes	XP_017273961.1	XP_017278464.1	NPHK	GASSL	DPHK	GASSL
*Austrofundulus limnaeus**	Annual killifish	Cyprinodontiformes	XP_013863753.1	XP_013861001.1	NPHK	GASSL	DPHK	GASSL
*Nothobranchius furzeri**	Turquoise killifish	Cyprinodontiformes	XP_015818782.1	XP_015820319.1	NPHK	GASSL	DPHK	GASSL
Xiphophorus couchianus	Monterrey platyfish	Cyprinodontiformes		XP_027858898.1			DPHK	GASSL
*Xiphophorus maculatus**	Southern platyfish	Cyprinodontiformes	XP_023197339.1	XP_023199771.1	NPHK	GASSL	DPHK	GASSL
Fundulus heteroclitus	Mummichog	Cyprinodontiformes	XP_021172482.1		NPHK	GASSL		
Poecilia reticulata	Guppy	Cyprinodontiformes		XP_008409743.1			DPHK	GASSL
Poecilia latipinna	Sailfin molly	Cyprinodontiformes		XP_014875252.1			DPHK	GASSL
Poecilia mexicana	Shortfin molly	Cyprinodontiformes		XP_014826206.1			DPHK	GASSL
Poecilia formosa	Amazon molly	Cyprinodontiformes		XP_007571850.1			DPHK	GASSL
Cyprinodon variegatus	Sheepshead minnow	Cyprinodontiformes		XP_015231204.1			DPHK	GASSL
Pimephales promelas	Fathead minnow	Cyprinodontiformes	CCI55629.1		DPHK	GASSL		
*Cynoglossus semilaevis**	Tongue sole	Pleuronectiformes	XP_008312881.1	NP_001281157.1	HPHK	GASSL	DPHK	GASSL
Scophthalmus maximus	Turbot	Pleuronectiformes		ADQ52413.1			DPHK	GASSL
Paralichthys olivaceus	Olive flounder	Pleuronectiformes	ACY69213.1	XP_019934595.1	DPHK	GASSL	DPHK	GASSL
*Oreochromis niloticus**	Nile tilapia	Cichliformes	XP_005448377.1	XP_003440542.2	NPHK	GASSL	DPHK	GASSL
Neolamprologus brichardi	Lyretail cichlid	Cichliformes		XP_006789718.1			DPHK	–
Pundamilia nyererei	Nyererei cichlid	Cichliformes	XP_005748571.1	XP_005737732.1	NPHK	GASSL	DPHK	GASSL
*Haplochromis burtoni**	Burton's mouthbrooder	Cichliformes	XP_005935524.1	XP_005941819.1	NPHK	GASSL	DPHK	GASSL
Astatotilapia calliptera	Eastern happy	Cichliformes	XP_026018663.1	XP_026032205.1	NPHK	GASSL	DPHK	GASSL
Maylandia zebra	Zebra mbuna	Cichliformes		XP_004563454.1			DPHK	GASSL
*Boleophthalmus pectinirostris**	Bluespotted mudhopper	Gobiiformes	XP_020779791.1	XP_020790489.1	NPHK	GASSL	DPHK	GASSL
Odontobutis obscura	Dark sleeper	Gobiiformes	QCC62347.1		NPHK	GASSL		

*Parambassis ranga**	Indian glassy fish	Ambassidae	XP_028287344.1	XP_028264353.1	NPHK	GASSL	DPHK	GASSL
*Stegastes partitus**	Bicolor damselfish	Pomacentridae	XP_008281503.1	XP_008279980.1	NPHK	GASSL	DPHK	GASSL
Amphiprion ocellaris	Ocellaris clownfish	Pomacentridae		XP_023149291.1			DPHK	GASSL
Acanthochromis polyacanthus	Spiny chromis	Pomacentridae		XP_022045651.1			DPHK	GASSL
Liparis tanakae	Tanaka's snailfish	Scorpaeniformes		TNN87891.1			DPHK	GASSL
Siniperca chuatsi	Chinese perch	Centrarchiformes		AVC70704.1			DPHK	GASSL
Oplegnathus fasciatus	Striped beakfish	Centrarchiformes	AHX37215.1		NPHK	GASSL		
*Notothenia coriiceps**	Black rockcod	Perciformes	XP_010773430.1	XP_010769312.1	NPHK	GASSL	DPHK	GASSL
*Cottoperca gobio**	Channel bull blenny	Perciformes	XP_029311984.1	XP_029289731.1	NPHK	GASSL	DPHK	GASSL
*Epinephelus coioides**	Orange-spotted grouper	Perciformes	AGC31487.1	ADA57613.1	NPHK	GASSL	DPHK	GASSL
Lateolabrax japonicus	Japanese sea bass	Perciformes	AXI69832.1		NPHK	GASSL		
*Larimichthys crocea**	Large yellow croaker	Perciformes	NP_001290316.1	NP_001290279.1	NPHK	GASSL	DPHK	GASSL
Collichthys lucidus	Spiny-head croaker	Perciformes	TKS89890.1	TKS73251.1	NPHK	GASSL	DPHK	GASSL
Miichthys miiuy	Chinese drum	Perciformes	AHB59737.1	AHB59742.1	NPHK	GASSL	DPHK	GASSL
Dicentrarchus labrax	European bass	Perciformes		AKG54863.1			DPHK	GASSL
Seriola dumerili	Greater amberjack	Perciformes	XP_022598320.1	XP_022609957.1	NPHK	GASSL	DPHK	GASSL
*Seriola lalandi dorsalis**	California yellowtail	Perciformes	XP_023267113.1	XP_023271214.1	NPHK	GASSL	DPHK	GASSL
*Echeneis naucrates**	Live sharksucker	Perciformes	XP_029382889.1	XP_029378048.1	NPHK	GASSL	DPHK	GASSL
*Lates calcarifer**	Barramundi	Perciformes	XP_018533852.1	XP_018519912.1	NPHK	GASSL	DPHK	GASSL
Labrus bergylta	Ballan wrasse	Perciformes		XP_020496966.1			DPHK	GASSL
Trachinotus ovatus	Pompano	Perciformes		ANA09016.1			DPHK	GASSL
*Anabas testudineus**	Climbing perch	Anabantiformes	XP_026207930.1	XP_026222283.1	NPHK	GASSL	DPHK	GASSL
Betta splendens	Siamese fighting fish	Anabantiformes		XP_029009386.1			DPHK	GASSL
Channa argus	Northern snakehead	Anabantiformes		ABK63482.1			DPHK	GASSL
*Mastacembelus armatus**	Tire track eel	Synbranchiformes	XP_026171852.1	XP_026168024.1	DPHK	GASSL	HPHK	GASSL
*Monopterus albus**	Asian swamp eel	Synbranchiformes	XP_020461964.1	XP_020474231.1	NPHK	GASSL	DPHK	GASSL
*Takifugu rubripes**	Japanese puffer	Tetraodontiformes	XP_003961331.1	XP_003967453.2	NPHK	GASSL	DPHK	GASSL
*Gadus morhua**	Atlantic cod	Gadiformes	XP_030195551.1	AJR33028.1	NPIK	GASSL	DPHK	GASSL
*Oryzias latipes**	Japanese rice fish	Beloniformes	XP_004080549.1		NPHK	GASSL		
*Oryzias melastigma**	Indian medaka	Beloniformes	XP_024140653.1	XP_024137304.1	NPHK	GASSL	DPHR	GASSL
Pygocentrus nattereri	Red-bellied piranha	Characiformes		XP_017578010.1			DPHK	GASSL
*Astyanax mexicanus**	Mexican tetra	Characiformes	XP_007248791.2	XP_007228105.2	NPHK	GASSL	DPHK	GASSL

[1]Total numbers of sequences used are 62 for IRF3 and 93 for IRF7.
[2]Forty-six species included in the phylogenetic analysis (Figure 2) are marked with *.
[3]Motif A color code used: DPHK in red, NPHK in blue, and all amino acid substitutions found are shown in black letters.
[4]Motif B color code used: GASSL in green, GVSSL in blue, and all amino acid substitutions found are shown in black letters. '-' indicates the motif sequence was not identified (including also a possible incomplete sequence).

Table S5: Protein sequences used for avian IRF7.[1]

Species[2]	Common name	Order	IRF7 Acc #	IRF7 motifs[3] A	IRF7 motifs[3] B
*Numida meleagris**	Helmeted guineafowl	Galliformes	XP_021259529.1	DPHK	GASSL
*Colinus virginianus**	Northern bobwhite	Galliformes	OXB73796.1	DPHK	GASSL
*Callipepla squamata**	Scaled quail	Galliformes	OXB53462.1	DPHK	GASSL
*Gallus gallus**	Red junglefowl (chicken)	Galliformes	NP_990703.1	DPHK	GASSL
*Coturnix japonica**	Japanese quail	Galliformes	XP_015719258.1	DPHK	GASSL
*Meleagris gallopavo**	Wild turkey	Galliformes	XP_003206360.1	DPHK	GASSL
*Anas platyrhynchos**	Mallard	Anseriformes	XP_027314523.1	DPHK	GASSL
Anser cygnoides domesticus	Swan goose	Anseriformes	XP_013029852.1	DPHK	GASSL
*Colius striatus**	Speckled mousebird	Coliiformes	KFP31281.1	DPHK	GASSL
*Amazona aestiva**	Turquoise-fronted amazon	Psittaciformes	KQK80008.1	DPHK	GASSL
*Melopsittacus undulatus**	Budgerigar	Psittaciformes	XP_012984533.1	DPHK	GASSL
*Mesitornis unicolor**	Brown mesite	Mesitornithiformes	KFQ37626.1	DPHK	GASSL
*Chaetura pelagica**	Chimney swift	Apodiformes	KFU88710.1	DPHK	GASSL
*Calypte anna**	Anna's hummingbird	Apodiformes	XP_008494285.1	DPHK	GASSL
*Haliaeetus leucocephalus**	Bald eagle	Accipitriformes	XP_010560879.1	DPHK	GASSL
*Aquila chrysaetos canadensis**	American golden eagle	Accipitriformes	XP_011594619.1	DPHK	GASSL
*Egretta garzetta**	Little egret	Pelecaniformes	XP_009638038.1	DPHK	GASSL
*Nipponia nippon**	Crested ibis	Pelecaniformes	XP_009468616.1	DPHK	GASSL
*Pygoscelis adeliae**	Adélie penguin	Sphenisciformes	XP_009321716.1	DPHK	GASSL
*Aptenodytes forsteri**	Emperor penguin	Sphenisciformes	XP_019326743.1	DPHK	GASSL
*Charadrius vociferus**	Killdeer	Charadriiformes	XP_009887688.1	DPHK	GASSL
*Calidris pugnax**	Ruff	Charadriiformes	XP_014812945.1	DPHK	GASSL
*Limosa lapponica baueri**	Bar-tailed Godwit	Charadriiformes	PKU46858.1	NPHK	GASSL
*Cuculus canorus**	Common cuckoo	Cuculiformes	KFO71916.1	DPHK	GASSL
*Acanthisitta chloris**	Rifleman	Passeriformes	XP_009078640.1	DPHK	GASSL
*Corvus brachyrhynchos**	American crow	Passeriformes	XP_017597090.1	DPHK	GASSL
Corvus cornix cornix	Hooded crow	Passeriformes	XP_010400558.1	DPHK	GASSL
*Taeniopygia guttata**	Zebra finch	Passeriformes	XP_002198341.1	DPHK	GASSL
Geospiza fortis	Medium ground finch	Passeriformes	XP_014161857.1	DPHK	GASSL
Lonchura striata domestica	Society finch	Passeriformes	XP_021410865.1	DPHK	GASSL
*Zonotrichia albicollis**	White-throated sparrow	Passeriformes	XP_005491662.1	DPHK	GASSL
*Serinus canaria**	Atlantic canary	Passeriformes	XP_018779912.1	DPHK	GASSL
Cyanistes caeruleus	Eurasian blue tit	Passeriformes	XP_023784659.1	DPHK	GASSL
*Pseudopodoces humilis**	Ground tit	Passeriformes	XP_005522631.2	DPHK	GASSL
Parus major	Great tit	Passeriformes	XP_018862540.1	DPHK	GASSL
*Sturnus vulgaris**	Common starling	Passeriformes	XP_014729049.1	DPHK	GASSL
*Ficedula albicollis**	Collared flycatcher	Passeriformes	XP_016153955.1	DPHK	GASSL
*Empidonax traillii**	Willow flycatcher	Passeriformes	XP_027760428.1	DPHK	GASSL
*Neopelma chrysocephalum**	Saffron-crested tyrant-manakin	Passeriformes	XP_027559024.1	DPHM	GASSL
*Corapipo altera**	White-ruffed manakin	Passeriformes	XP_027498314.1	DPHM	GASSL
*Lepidothrix coronata**	Blue-crowned manakin	Passeriformes	XP_017668837.1	DPHM	GASSL
Manacus vitellinus	Golden-collared manakin	Passeriformes	XP_017937494.1	DPHM	GASSL
*Pipra filicauda**	Wire-tailed manakin	Passeriformes	XP_027583518.1	DPHM	GASSL
Falco cherrug	Saker falcon	Falconiformes	XP_014138356.1	DPHK	GASSL
*Falco peregrinus**	Peregrine falcon	Falconiformes	XP_013153706.1	DPHK	GASSL
*Columba livia**	Rock dove	Columbiformes	XP_021152373.1	DPHK	GASSL
*Patagioenas fasciata monilis**	Band-tailed pigeon	Columbiformes	OPJ73738.1	DPHK	GASSL

Tinamus guttatus	White-throated tinamou	Tinamiformes	XP_010214274.1	DPHK GASSL
Nothoprocta perdicaria	Chilean tinamou	Tinamiformes	XP_025890574.1	DPHK GASSL
*Apteryx rowi**	Okarito kiwi	Apterygiformes	XP_025914875.1	DPHK GASSL

[1]Total number of IRF7 sequences used is 50.
[2]Forty species included in the phylogenetic analysis (Figure S5) are marked with *.
[3]Motif A color code used: DPHK in red, NPHK in blue, and all amino acid substitutions found are shown in black letters. Motif B (GASSL) is shown in green.

Table S6: Sequence variations found in the GASSL motif.

	Position in the motif[1]				
	**1 [G]**	**2 [A]**	**3 [S]**	**4 [S]**	**5 [L]**
Total # of variations (IRF3:IRF7)	0	104 (0:104)	2 (1:1)	1 (1:0)	10 (6:4)
Variations in IRF3			T (1/59 ray-finned fish)	P (1/125 mammals)	R (1/59 ray-finned fish)<br>P (1/59 ray-finned fish)<br>M (4/125 mammals)
Variations in IRF7		V (87/120 mammals, 1/13 reptiles)<br>T (7/120 mammals, 4/13 reptiles)<br>L (1/120 mammals)<br>I (2/120 mammals)<br>F (1/13 reptiles)<br>C (2/13 reptiles)	T (1/90 ray-finned fish)		I (1/5 amphibians, 2/90 ray-finned fish)<br>W (1/120 mammals)

[1]Consensus amino acid for each position is shown in the square brackets.

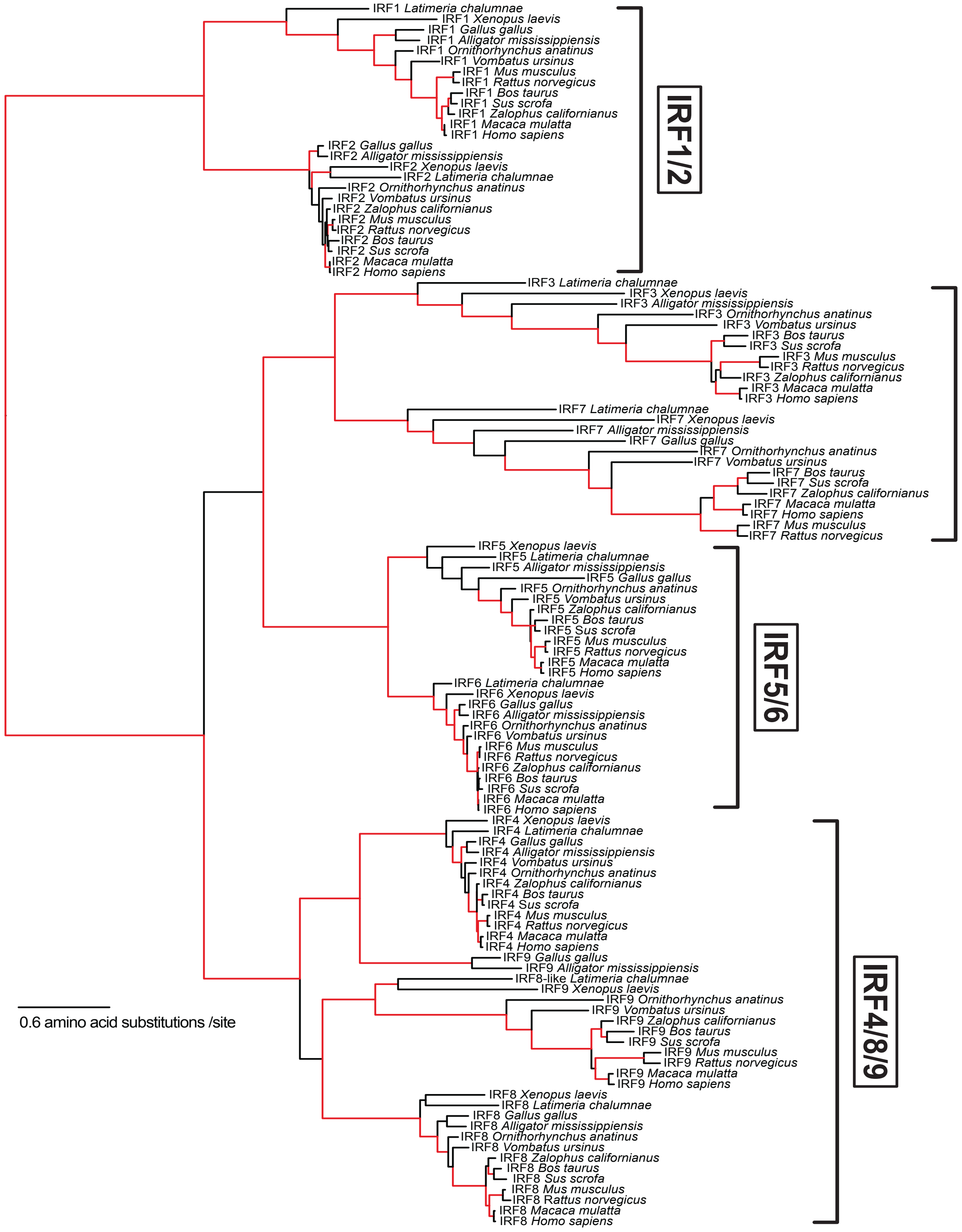

Figure S1. The maximum likelihood phylogeny of the IRF protein family. For each IRF group, protein sequences from 13 representative vertebrate species are included (except for IRF3 where birds have no IRF3). The accession numbers for the sequences used are listed in Table S1. Internal branches supported by the bootstrap values equal to or higher than 70% are shown in red.

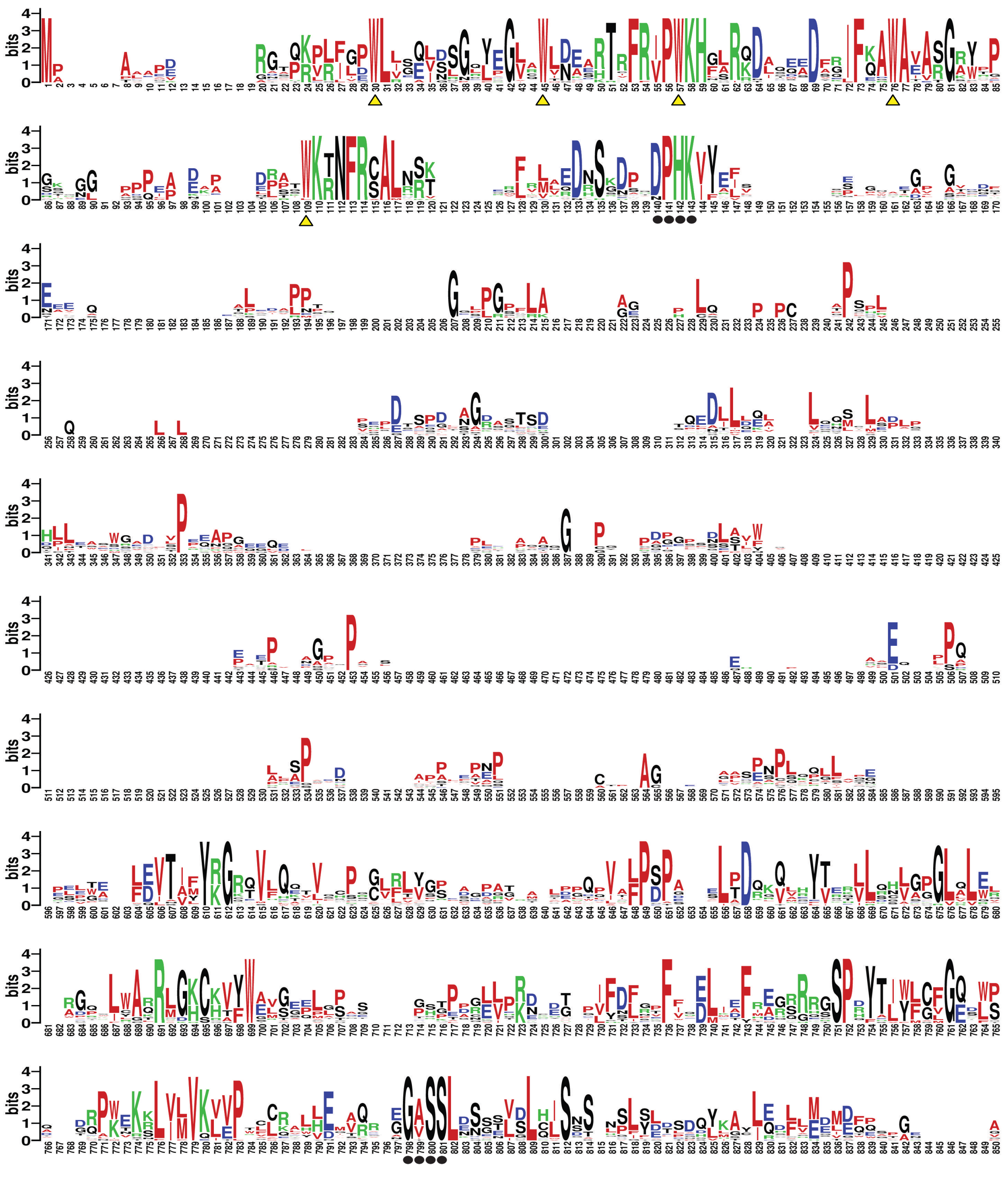

Figure S2. Sequence conservation of the IRF3/7 protein family. The amount of sequence conservation for each position is represented by sequence logo. The overall height of the stack of letters indicates the sequence conservation at that position. The height of symbols within each stack indicates the relative frequency of each amino acid at that position. Colors used for different amino acids are as follows: green (K, R, H), blue (D and E), red (A, V, L, I, P, W F, M), and black for all others. A total of 25 protein sequences (12 of IRF3 where birds have no IRF3 and 13 of IRF7) are included in the multiple sequence alignment. The accession numbers for the sequences used are listed in Table S1. The five conserved tryptophans (Ws) are pointed with yellow triangles. The two conserved motif regions (A and B) are indicated by black dots.

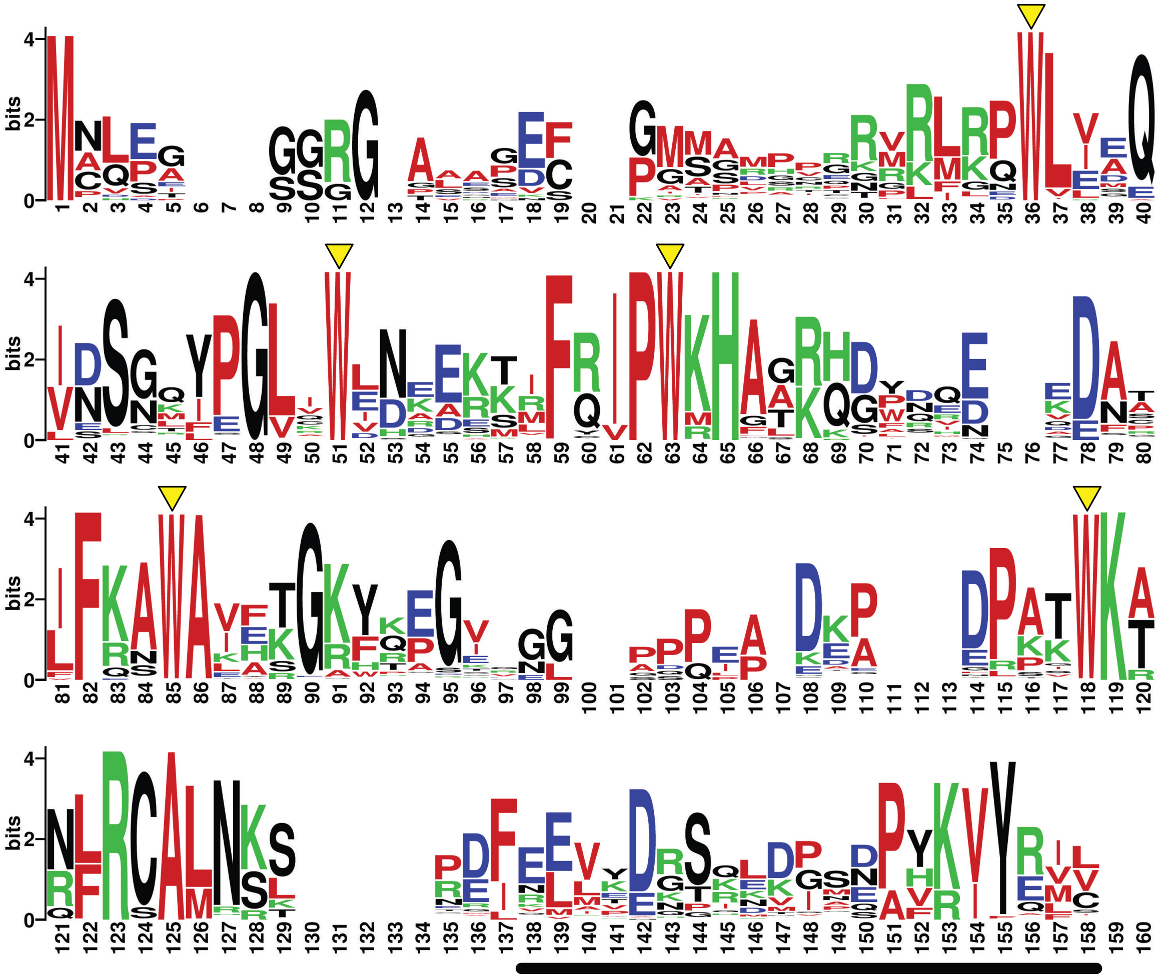

Figure S3. Sequence conservation of the DBD region of the IRF protein family. The amount of sequence conservation for each position is represented by sequence logo. The overall height of the stack of letters indicates the sequence conservation at that position. The height of symbols within each stack indicates the relative frequency of each amino acid at that position. Colors used for different amino acids are as follows: green (K, R, H), blue (D and E), red (A, V, L, I, P, W F, M), and black for all others. A total of 116 protein sequences (13 each for the nine IRF families, except for IRF3 where birds have no IRF3) are included in the multiple sequence alignment. The accession numbers for the sequences used are listed in Table S1. The five conserved tryptophans (Ws) are pointed with yellow triangles. The sequence logos specific to each IRF group were produced from the region marked with a thick black bar and presented in Figure 1b and c.

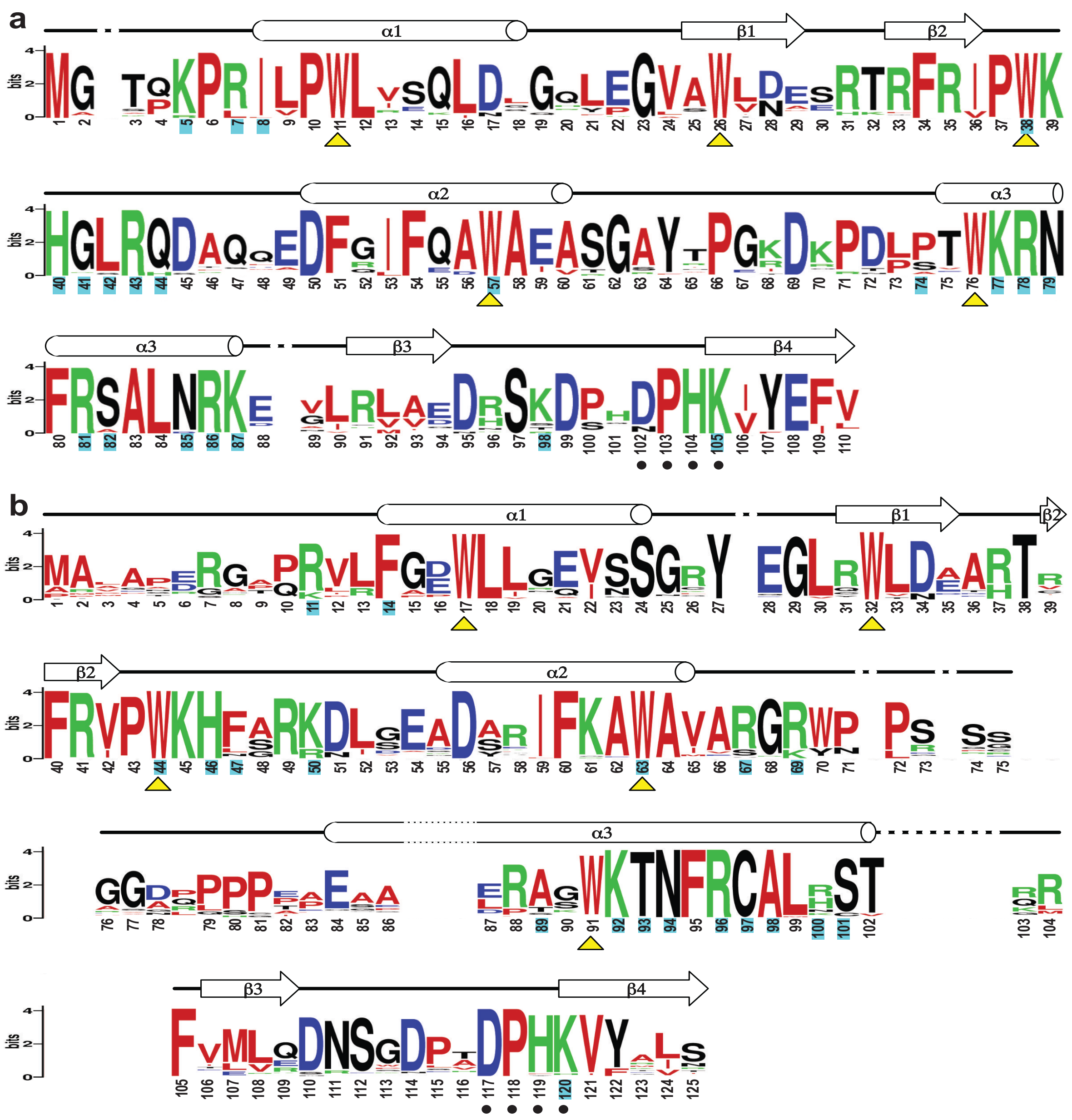

Figure S4. Sequence conservation of the DBD regions of the IRF3 (a) and IRF7 (b) subfamilies. The amount of sequence conservation for each position is represented by sequence logo. The overall height of the stack of letters indicates the sequence conservation at that position. The height of symbols within each stack indicates the relative frequency of each amino acid at that position. Colors used for different amino acids are as follows: green (K, R, H), blue (D and E), red (A, V, L, I, P, W F, M), and black for all others. Multiple sequence alignments were generated using 66 IRF3 and 65 IRF7 sequences listed in Tables S2 and S3. The five conserved tryptophans (Ws) are pointed with yellow triangles. The DPHK motif is marked with black dots. The secondary structure as well as the protein-DNA contact position (numbers in blue background) information is taken from Panne *et al*. (2007). The position numbers are based on the human IRF3 and IRF7 proteins (UniProt accession #: Q14653 and Q92985, respectively).

Reference:

Panne, D., Maniatis, T., and Harrison, S. C. (2007) An atomic model of the interferon-beta enhanceosome. *Cell* **129**:1111-1123.

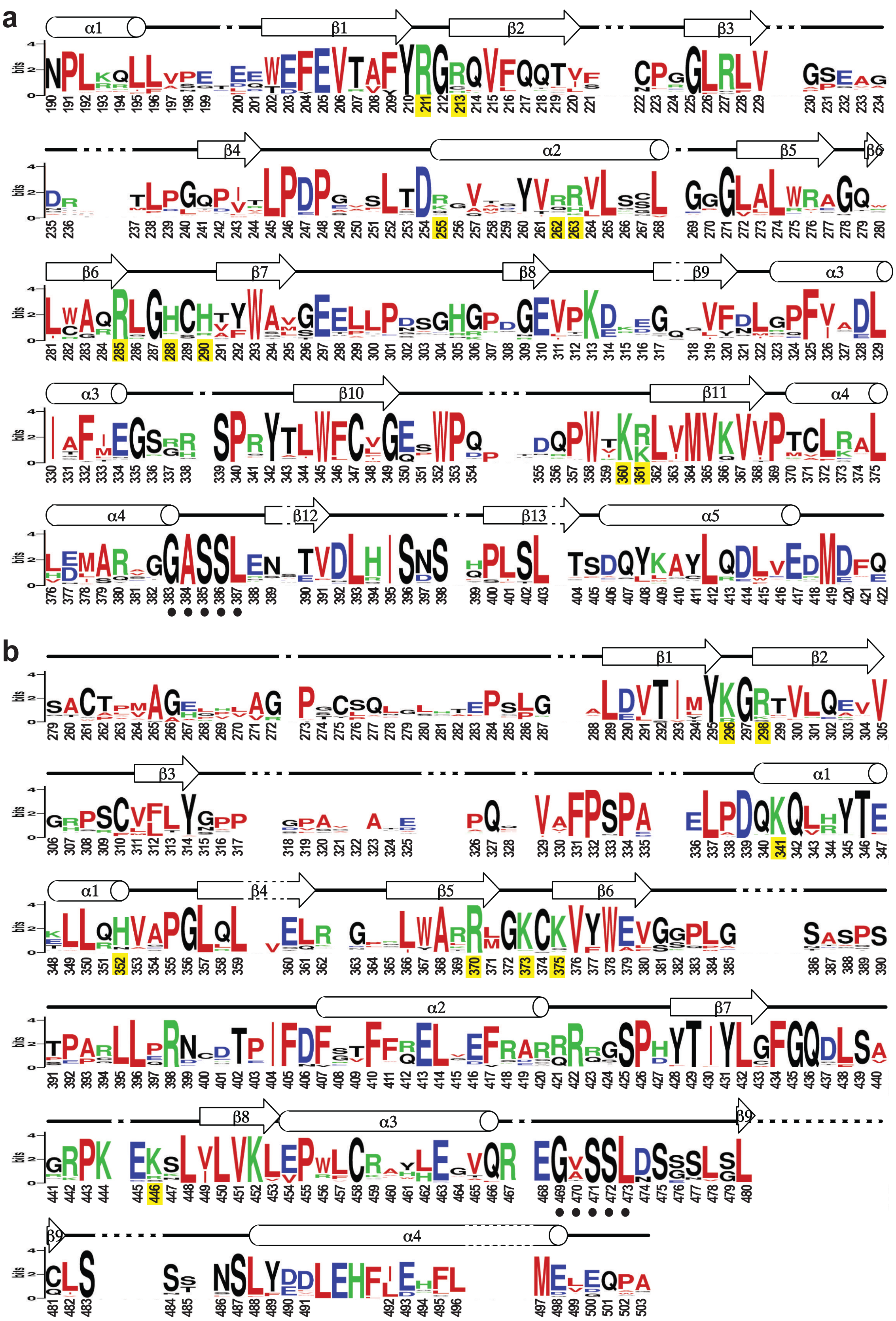
a
α1
β1
β2
β3
β4
α2
β5
β6
β7
β8
β9
α3
β10
β11
α4
β12
β13
α5
bits
b
β1
β2
β3
α1
β4
β5
β6
α2
β7
β8
α3
β9
α4

Figure S5. Sequence conservation of the C-terminal regions of the IRF3 (a) and IRF7 (b) subfamilies. The amount of sequence conservation for each position is represented by sequence logo. The overall height of the stack of letters indicates the sequence conservation at that position. The height of symbols within each stack indicates the relative frequency of each amino acid at that position. Colors used for different amino acids are as follows: green (K, R, H), blue (D and E), red (A, V, L, I, P, W F, M), and black for all others. Multiple sequence alignments were generated using 66 IRF3 and 65 IRF7 sequences listed in Tables S2 and S3. The GASSL and GVSSL motifs are marked with black dots. The secondary structure information for IRF3 (a) is taken from Qin *et al.* (2003). For IRF7 (b), the secondary structure was predicted using PSIPRED 4.0 (Buchan and Jones, 2019). The positions of the ten positively charged amino acids identified to form the basic surface by Qin *et al.* (2003) are marked with yellow for IRF3 (a). The corresponding positions that have positively charged amino acids are also marked with yellow in IRF7 (b) The position numbers are based on the human IRF3 and IRF7 proteins (UniProt accession #: Q14653 and Q92985, respectively).

References:

Buchan, D. W. A. and Jones, D. T. (2019) The PSIPRED Protein Analysis Workbench: 20 years on. *Nucleic Acids Res*. **47**: W402-W407.

Qin, B. Y., Liu, C., Lam, S.S., Srinath, H., Delston, R., Correia, J.J., Derynck, R., Lin, K. (2003) Crystal structure of IRF-3 reveals mechanism of autoinhibition and virus-induced phosphoactivation. *Nat Struct Biol.* **10**: 913-921.

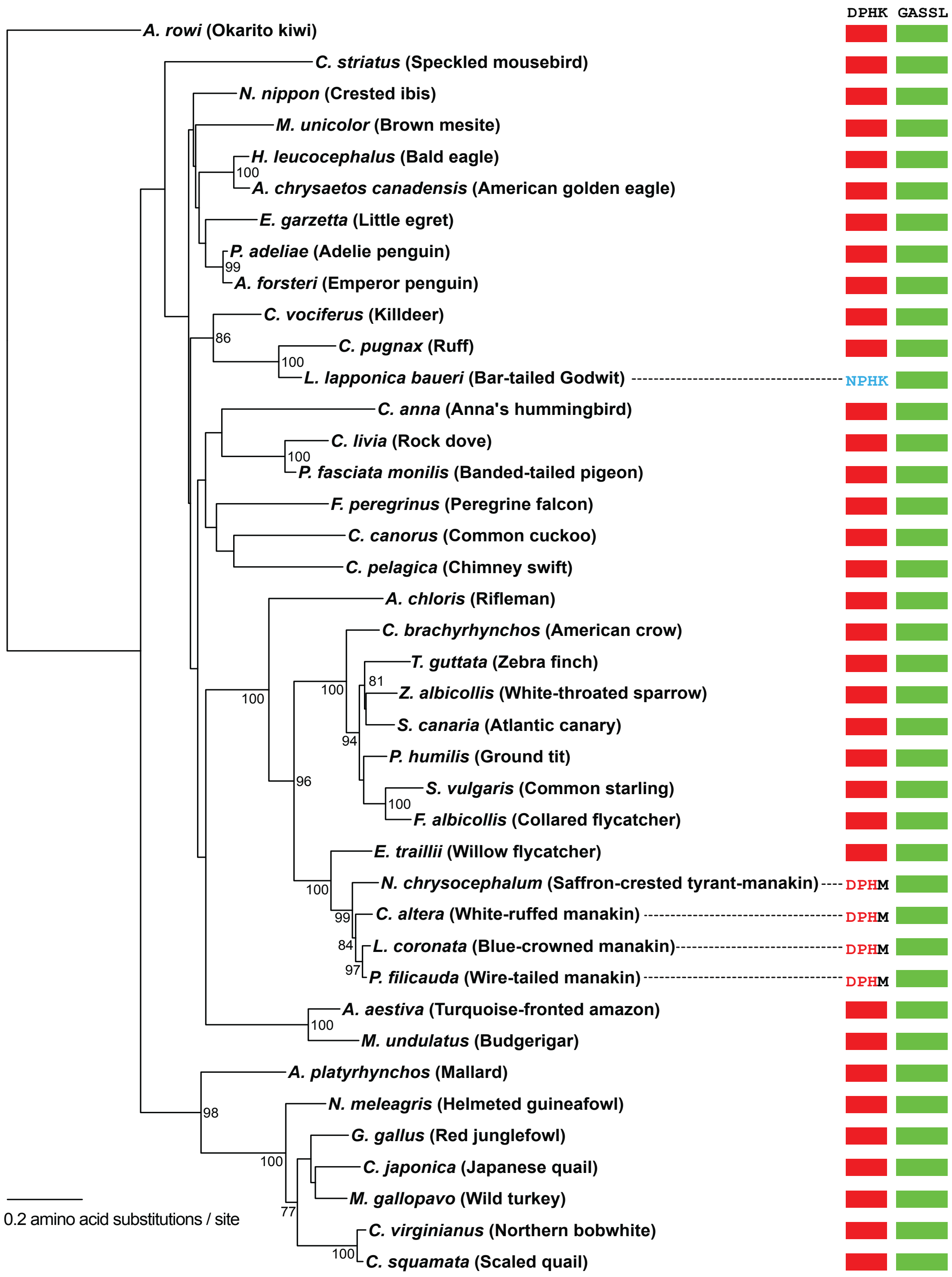

Figure S6. The maximum likelihood phylogeny of the avian IRF7 proteins and the amino acid sequences found in the DPHK and GASSL motifs. Forty avian IRF7 protein sequences are included. The accession numbers for the sequences used are listed in Table S5. *Apteryx rowl* (Okarito kiwi; Apterygiformes) was used as the outgroup. Numbers shown at nodes are the bootstrap supporting values (%). Only those equal to or higher than 70% are shown. For each IRF7 protein sequence, the existence of the DPHK and GASSL motifs are shown with red and green rectangles. When variant sequences are found, actual amino acid sequences are shown.
